# Long non-coding RNAs involved in the regulatory network during porcine pre-implantation embryonic development and iPSC induction

**DOI:** 10.1038/s41598-018-24863-5

**Published:** 2018-04-27

**Authors:** Liang Zhong, Haiyuan Mu, Bingqiang Wen, Wei Zhang, Qingqing Wei, Ge Gao, Jianyong Han, Suying Cao

**Affiliations:** 1 The Animal Science and Technology College, Beijing University of Agriculture, Beijing, China; 2 State Key Laboratory for Agrobiotechnology, College of Biological Sciences, China Agricultural University, Beijing, China; 3 State Key Laboratory of Protein and Plant Gene Research, College of Life Sciences, Center for Bioinformatics, Peking University, Beijing, China; 4 Beijing Key Laboratory of Traditional Chinese Veterinary Medicine, Beijing University of Agriculture, Beijing, China

## Abstract

Long non-coding RNAs (lncRNA) play a key role in the orchestration of transcriptional regulation during development and many other cellular processes. The importance of the regulatory co-expression network was highlighted in the identification of the mechanism of these processes in humans and mice. However, elucidation of the properties of porcine lncRNAs involved in the regulatory network during pre-implantation embryonic development and fibroblast reprogramming to induced pluripotent stem cell (iPSC) has been limited to date. Using a weighted gene co-expression network analysis, we constructed the regulatory network and determined that the novel lncRNAs were functionally involved in key events of embryonic development during the pre-implantation period; moreover, reprogramming could be delineated by a small number of potentially functional modules of co-expressed genes. These findings indicate that lncRNAs may be involved in the transcriptional regulation of zygotic genome activation, first lineage segregation and somatic reprogramming to pluripotency. Furthermore, we performed a conservation and synteny analysis with the significant lncRNAs involved in these vital events and validated the results via experimental assays. In summary, the current findings provide a valuable resource to dissect the protein coding gene and lncRNA regulatory networks that underlie the progressive development of embryos and somatic reprogramming.

## Introduction

Long non-coding RNA (lncRNA) is a type of non-protein coding transcript longer than 200 nucleotides that represents a large and diverse class of non-coding RNA. The advances of past several years in RNA sequencing (RNA-Seq) and computational methodology have enabled an unprecedented analysis of these transcripts. Genome-wide analyses have identified more than 9,000 human long intergenic noncoding RNAs (lincRNA)^1–4^ and more than 10,000 long noncoding transcripts in mice^5,6^. Recently, thousands of lncRNAs have been identified in other model organisms as well as domesticated animals, such as *C. elegans*^7^, *Drosophila*^8^, zebrafish^9,10^, chicken^11^, cattle^12–14^ and pigs^15–18^. However, only a few lncRNAs have been functionally annotated, and the majority remains to be characterized. Several lncRNA databases have been created to provide an integrative annotation of lncRNAs^19–23^.

The pig is not only a commercial domesticated animal but also an ideal experimental animal for medical and preclinical models that shares similar characteristics with humans in physiology, anatomy and the occurrence of disease^24^. While the other model animals, such as mice, cannot exactly be used to evaluate the long-term effects of cell differentiation or cell therapy, and human research may result in ethical issues in the developmental process^25,26^. The difference in pre-implantation development in mice and humans has made pigs invaluable for basic investigations in which it may serve as an excellent model system to investigate developmental biology, reprogramming and embryonic cell fate commitment. However, to date, no authentic porcine embryonic stem cells have been derived; thus, a comprehensive analysis of pre-implantation embryos and induced pluripotent stem cells (iPSCs) would facilitate the understanding of the mechanism of pluripotency in pigs. Moreover, iPSCs provide tremendous opportunities for regenerative medicine. Many of the molecular mechanisms that underlie tumorigenesis and self-renewal in iPSCs have been elucidated in recent years.

Embryogenesis is the process in which an embryo forms and develops, and it is initiated by the fertilization of the oocyte with a sperm cell. The zygote subsequently undergoes several rapid rounds of mitotic division without significant growth (a process referred to as the cleavage stage) and cellular differentiation, which lead to the development of a multicellular embryo. Interestingly, porcine transcriptome data have indicated that zygotic genome activation (ZGA) occurs during the period from 4-cell stage to 8-cell stage, and there are shared molecular determinants and pathways among pigs, mice and humans involved in first lineage segregation and primitive endoderm differentiation^27^. However, little is known regarding the potential signaling elements that constitute the regulatory network involved in porcine pre-implantation embryo development, especially the interaction with non-coding RNA during this vital period.

An investigation of the expression patterns of lncRNAs and mRNA at a particular cell-stage may identify novel functional connections and expand the mechanistic knowledge of the development process. In this study, we report the genome-wide characterization of iPSCs and all stage-embryonic lncRNAs and define a stringent set of 207 (563 transcripts) novel lncRNA genes from RNA-seq data of porcine embryos. We validated our data set using genomic features of lncRNA, including transcript length, exon number, evolutionary conservation and spatiotemporal expression specificity. A weighted gene co-expression network analysis indicated that lncRNAs are expressed in a strong developmental stage-specific manner, and many lncRNAs are associated with developmental regulatory genes. Furthermore, to derive authentic porcine embryonic stem cells, an intrinsic issue was whether the lncRNAs of piPSCs are regulated by identical or comparable mechanisms in the blastocyst stage. Our genome-wide annotation of embryonic lncRNAs may improve our understanding of the molecular mechanisms that underlie porcine embryogenesis as well as reprogramming and provide several regulatory networks during developmental process.

## Results

### Pipeline of lncRNA identification

**Figure 1:**
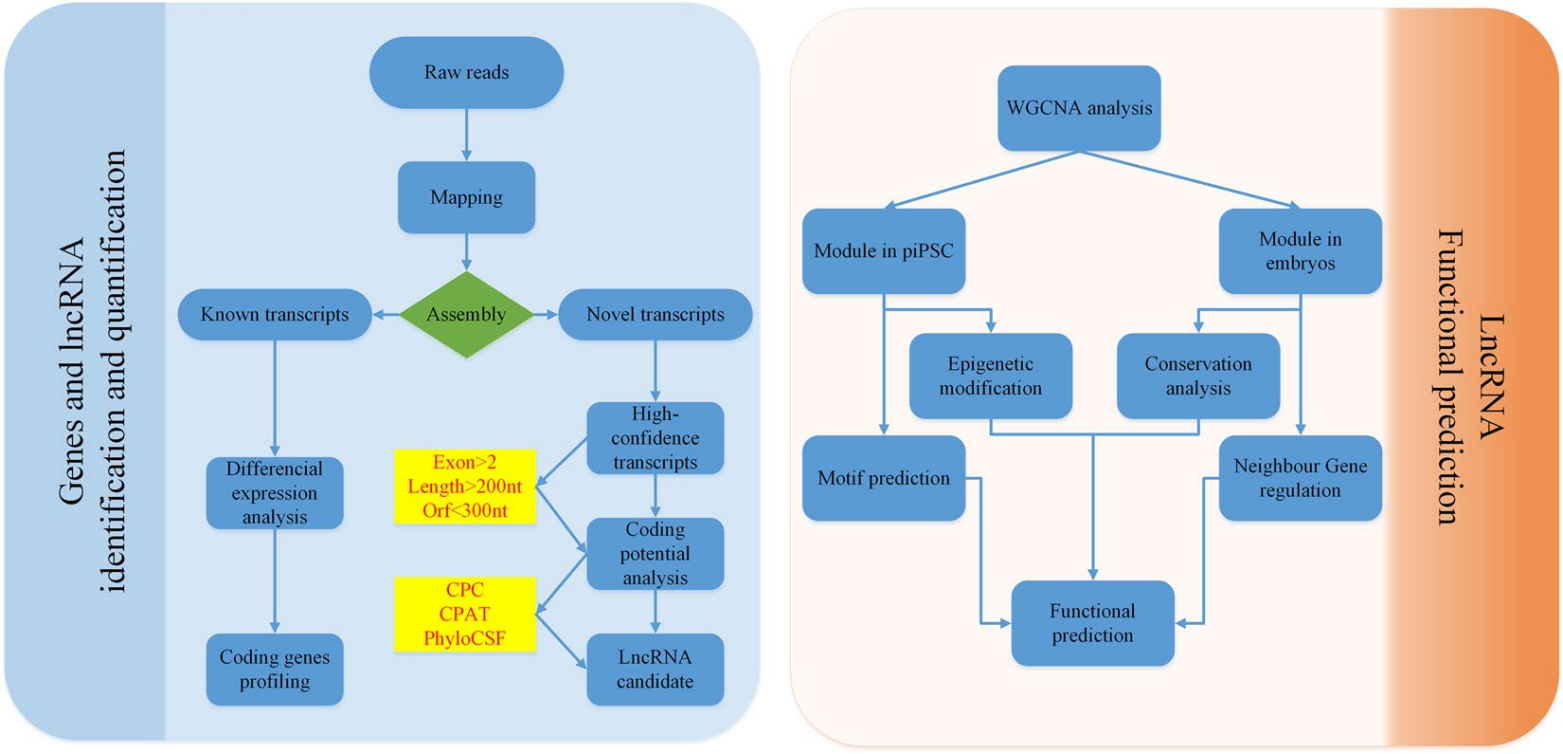
Pipeline for the identification of novel potentially functional lncRNAs in porcine pre-implantation embryos and iPSCs. The left figure is the pipeline for identification of lncRNAs from transcriptomes of porcine pre-implantation embryos and iPSCs. Transcripts were classified as known protein-coding genes and novel transcripts. The novel high-quality transcripts were subsequently filtered by sequence feature, coding potential, phylogenetic conservation as well as dissimilarity to known protein domains, and the remaining transcripts were defined as lncRNAs. The right figure is the pipeline for investigation of functional lncRNAs. To identify potentially functional lncRNAs during porcine embryo development and reprogramming to pluripotency, a co-expression network was constructed with protein coding genes and lncRNAs by WGCNA. The lncRNAs in modules that correlated with important embryonic stage and cell type were chosen to perform epigenetic modification, conservation analysis and experimental verification. Finally, the potentially functional lncRNAs in specific regulatory co-expression network were elucidated.

To identify potentially functional lncRNAs and the regulatory network involved in porcine pre-implantation embryos and iPSCs, we first aligned clean reads to the porcine genome (susScr2) using TopHat and assembled them subsequently into 621,085 transcripts to form 227,625 loci in all samples using Cufflinks, which include 27 RNA-seq samples composing of oocyte, 7 consecutive embryonic stages and iPSC. Each of the cell type has biological replicates. Besides, porcine fibroblasts have been sequenced to compare with iPSC. To obtain high-quality novel transcripts, we used Ensembl, NCBI RefSeq and UCSC gene annotations to eliminate known genes and removed the lowly expressed transcripts. The expression level of the lncRNAs was relatively low; therefore, a cutoff threshold was defined for the novel transcripts. According to the peak of Gaussian distribution at the kernel density estimation, we selected transcripts to cut off the FPKM (fragments per kilobaseof exon per million fragments mapped) of all samples (Supplementary Fig. 1). Then we obtained the highly confidential transcript which could be used to identify lncRNA with a stringent pipeline. Thus, the remaining transcripts were defined as novel lncRNAs and were used for the downstream functional analysis (Fig. 1).

### Transcriptome profiling of porcine pre-implantation embryos and iPSCs

We used porcine oocytes, *in vivo* embryos, somatic cell nuclear transfer (SCNT) embryos, fibroblasts, and iPSCs to conduct RNA-seq. iPSCs were generated from fibroblasts and passaged 20 times. The embryos were at several critical stages of pre-implantation development, namely, zygote, 2-cell, 4-cell, 8-cell, morula and blastocyst. To elucidate the differences between the inner cell mass (ICM) and trophectoderm (TE), we physically segregated the TE and ICM. All embryo samples were collected according to stringent morphological criteria and sequenced.

First, we analyzed the known coding genes in each sample. Approximately 12,000 to 14,000 known Ensembl genes were detected (FPKM >0.1) out of 20,459 Ensembl genes (release-66). On average, the detected expression genes represented 14,275 (69.7%); thus, more than half of the known porcine transcripts were expressed, which suggests that our transcriptomes were reliable for different downstream analysis (Supplementary Fig. 2).

**Figure 2:**
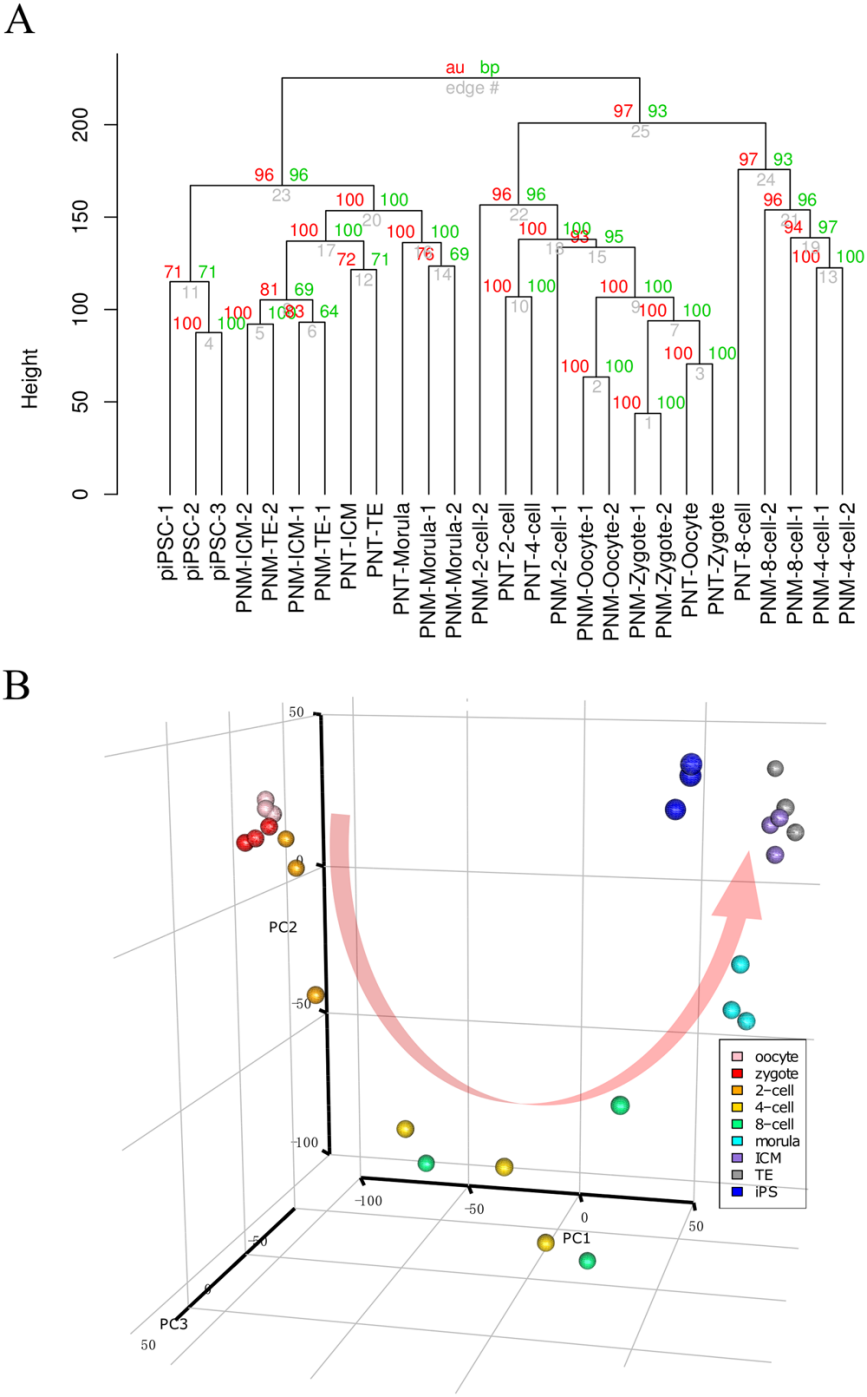
Global profiling of the porcine pre-implantation embryo and iPSC transcriptome. (**A**) Hierarchical cluster analysis of known genes. WPGMA (Weighted Pair Group Method with Averaging) is used as the agglomeration method. AU (Approximately Unbiased) in red color represents p-value and BP (Bootstrap Probability) value in green color. AU p-value is computed by multiscale bootstrap resampling method. The prefixes PNM and PNT of embryonic samples represent porcine *in vivo* embryos and porcine somatic cell nuclear transfer (SCNT) embryo samples, respectively. (**B**) Principal component analysis (PCA) of the porcine pre-implantation embryo and iPSC transcriptome. PC1, PC2 and PC3 represent the top three dimensions of the contribution of gene expression among these samples, which account for 48.9%, 10.4% and 8.0%, respectively.

To investigate the relationships among the developmental stages, we performed hierarchical clustering and a principal component analysis (PCA) of the known genes individual embryonic stages and iPSCs **(**Fig. 2A,B**)**. A distinct cluster formed as a result of different pre-implantation embryonic stages and cell types. There were 2 clusters that were mainly separated by the 8-cell stage, a group of earlier than the 8-cell stage samples in which oocyte, zygote and 2-cell stages were more similar to each other and a second group of 4-cell and 8-cell stage samples. The consecutive stages agglomerated together in the analysis as expected, and from 4-cell stage to 8-cell stage embryos were separated from early samples as a result of degradation of maternal mRNA after fertilization and ZGA between the 4-cell and 8-cell stages, which has been previously reported^27^. During the morula and blastocyst stages, blastomeres began to differentiate, which resulted in the first cell fate determination in embryogenesis, and progressively segregated into the ICM and TE. Furthermore, piPSCs were similar to porcine blastocysts, which could be explained by the pluripotency during the lineage segregation, and a small number of blastomeres would develop into the pluripotent ICM (Supplementary Fig. 3**)**. However, another notable difference occurred between the *in vivo* embryos and SCNT embryos; there was a lag during the embryonic development to the 8-cell stage, particularly at the 4-cell and 8-cell stages, because the reconstruction of the alternated gene expression of the SCNT embryos has a critical impact on embryogenesis^28^.

The results that the same embryonic stage exhibits a greater similarity than between different stages were also supported by the PCA plot, which indicates that the embryos in a given developmental stage were gathered closely and the continuous stage also presented the trends for embryogensis. Intriguingly, the samples of 4-cell and 8-cell stages were dispersed in the PCA plot though they were relatively similar in the hierarchical cluster results, which sugguest that complicated regulation emerged during that period. Porcine ZGA occurs at the 4-cell stage, and as many as 1791 genes exhibited differential expression between the 4-cell stage and 8-cell stage. Therefore, we suggest that the 4-cell and 8-cell stages play an important role in the course of pre-implantation embryogenesis.

### Genomic features and stage-specific expression of lncRNAs

**Figure 3:**
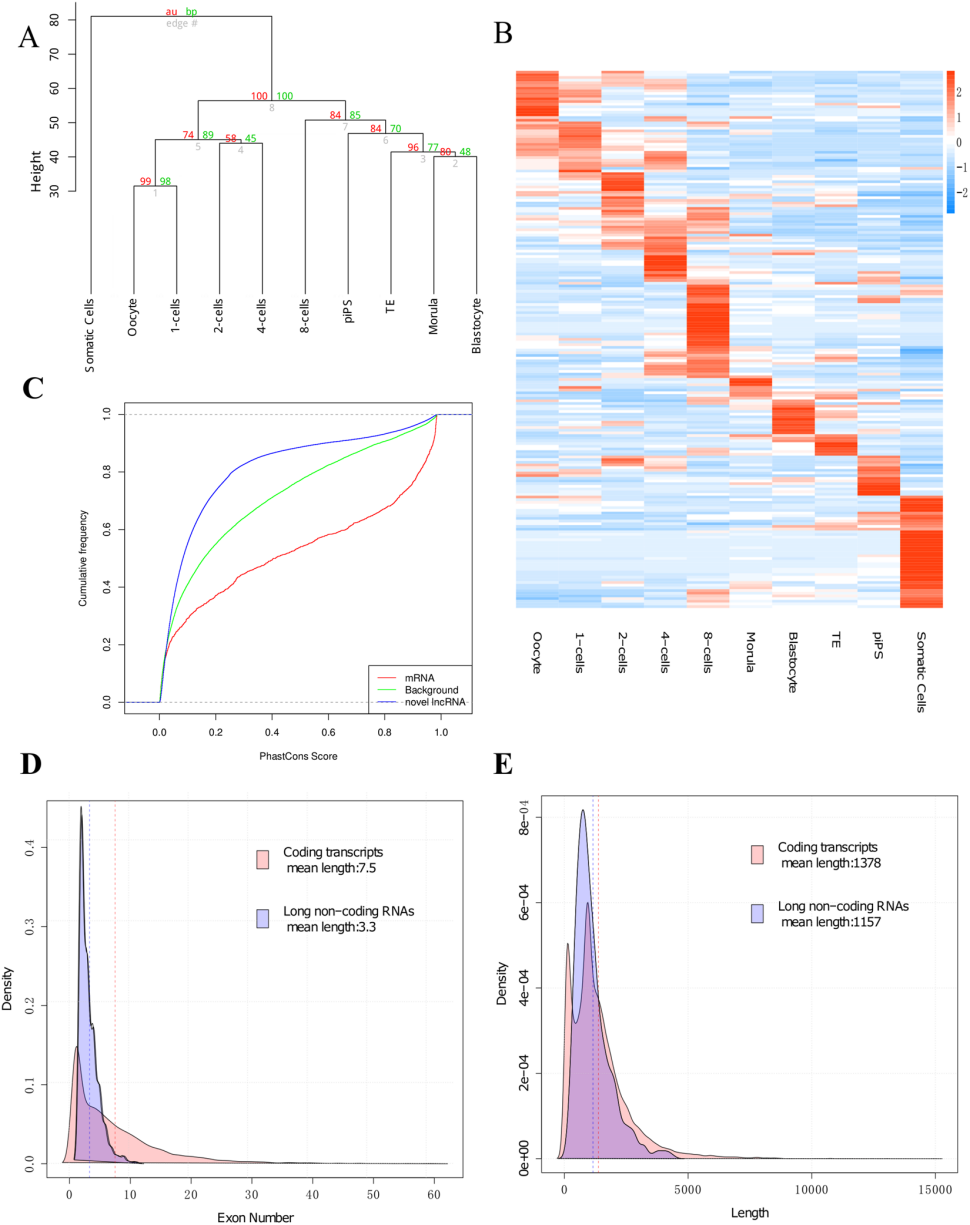
Genomic features and stage-specific expression of lncRNAs. (**A**) Hierarchical cluster analysis of novel long non-coding RNAs. WPGMA is used as the agglomeration method. AU (Approximately Unbiased) in red color represents p-value and BP (Bootstrap Probability) value in green color. AU p-value is computed by multiscale bootstrap resampling method. We combined all samples in the same stage for analysis. (**B**) Heatmap of stage-specific expression of the novel lncRNAs, which was calculated by the Jensen-Shannon distance. (**C**) The cumulative curve of the average phastCons score of the novel lncRNAs (blue), mRNA (red) and the genomic background (green). Genomic background was randomly selected from the whole genome. (**D**) and (**E**) Length and exon number distribution between porcine lncRNAs and protein coding genes; the blue portion represents the lncRNAs, and the pink portion represents the protein coding genes.

Previous studies have shown that the expression levels of lncRNAs occur in a cell-type-specific manner and are significantly lower than protein coding transcripts; moreover, lncRNAs were shorter and less conserved than protein coding transcripts^1,6^. Those reports have suggested that lncRNAs have temporal- and spatial-specific expression, which plays important roles during embryogenesis^10^. However, lncRNAs and their biologically relevant processes in porcine embryonic development are poorly understood. Deciphering the repertoire and expression profiles of lncRNAs, particularly the temporal and spatial expression patterns, would provide a vital step towards a better understanding of the gene regulatory network within the developmental processes. We performed a hierarchical clustering analysis for lncRNAs **(**Fig. 3A**)**; however, in contrast to the protein coding genes, the dendrogram of embryonic samples was mainly classified into 2 groups, which were separated by the 4-cell to 8-cell stage. Potentially as a result of ZGA, the 4-cell to 8-cell stage embryos were more inclined to vary in the expression of protein coding genes and lncRNAs.

To identify the stage-specific expression of lncRNAs, we performed differential expression analysis for pairwise comparison of all 10 type of samples. Furthermore, we calculated the temporal specificity scores using the Jensen-Shannon distance method **(**Fig. 3B**)**. The 4-cell and 8-cell stage embryos were the most variable in the expression of lncRNAs, and there were 17 up-regulated lncRNAs (log2FC of 8-cell versus 4-cell >1, adjusted-*P* < 0.05) in contrast to only 7 down-regulated lncRNAs (log2FC of 8-cell versus 4-cell <−1, adjusted-*P* < 0.05) from the 4-cell to 8-cell stage. Interestingly, we obtained the opposite results in the expression patterns of the protein coding genes **(**Supplementary Tables S3 and S4**)**; the 4-cell and 8-cell stage embryos had more down-regulated differentially expressed genes (963 genes, log2FC <−1 and adjusted-*P* < 0.05) than up-regulated genes (828 genes, log2FC >1 and adjusted-*P* < 0.05), which suggests that the degradation of maternal transcripts and zygotic genome activation tend to occur in parallel within developmental activities.

In our study, we found that lncRNA show less conversation than mRNA even in randomly selected genomic background, which display with curve of accumulative PhastCons scores in Fig. 3C. We also determined that the porcine novel lncRNAs exhibit a fewer number of exons and are shorter in sequence length compared with protein coding genes. As indicated in Fig. 3D,E, the distribution of the transcript exon number and length indicates that mRNA has 7.5 exons and 1378 nucleotides in length on average, whereas lncRNA has 3.3 exons and 1157 nucleotides in length on average; these findings indicate a significant difference between mRNA and lncRNAs (exon number: 7.5 versus 3.3, respectively, *P* = 3.798 × 10^−29^; length: 1378 versus 1157, respectively, *P* = 1.833 × 10^−8^, Welch two-sample t-test). Interestingly, the porcine lncRNAs were similar to the genomic features of human (approximately 1000 nt in length and 2.9 exons on average), mouse (approximately 1300 nt in length and 2.3 exons on average) and zebrafish (approximately 1113 nt in length and 2.8 exons on average) lncRNAs according to the NONCODEv4 database^23^. The similarity of the characterization of multi-species lncRNA suggests the porcine lncRNA results were close to those in other model organisms.

### Construction of a regulatory network using WGCNA

We subsequently applied a weighted gene co-expression network analysis (WGCNA)^29^ to investigate the co-expression relationships between the protein coding genes and lncRNAs that were associated with pre-implantation embryonic development and somatic cell reprogramming to pluripotency at a system level. WGCNA identified 25 significant discrete modules of co-expressed genes and lncRNAs (Supplementary Figs 4–6). We focused on 11 of 25 modules that were highly correlated (r > 0.6, *P* < 0.05) and associated with embryo development as well as stem cell maintenance in specific stages. Most notably, the “darkred” (n = 98, r = 0.82, *P* = 2 × 10^−7^) and “purple” (n = 184, r = 0.88, *P* = 2 × 10^−9^) modules were involved in the transition from oocyte to zygote, and the “blue” (n = 713, r = 0.95, *P* = 8 × 10^−14^) module was involved in reprogramming to pluripotency. However, for the remaining significant modules that participated in the pre-implantation embryo development, we determined that several modules showed coordinated changes across several successive stages. These findings suggested that the identified coordinated modules were inclined to relate specifically to the developmental events of pre-implantation, such as ZGA and lineage segregation.

### Regulatory network of zygotic genome activation during 4-cell and 8-cell stages

**Figure 4:**
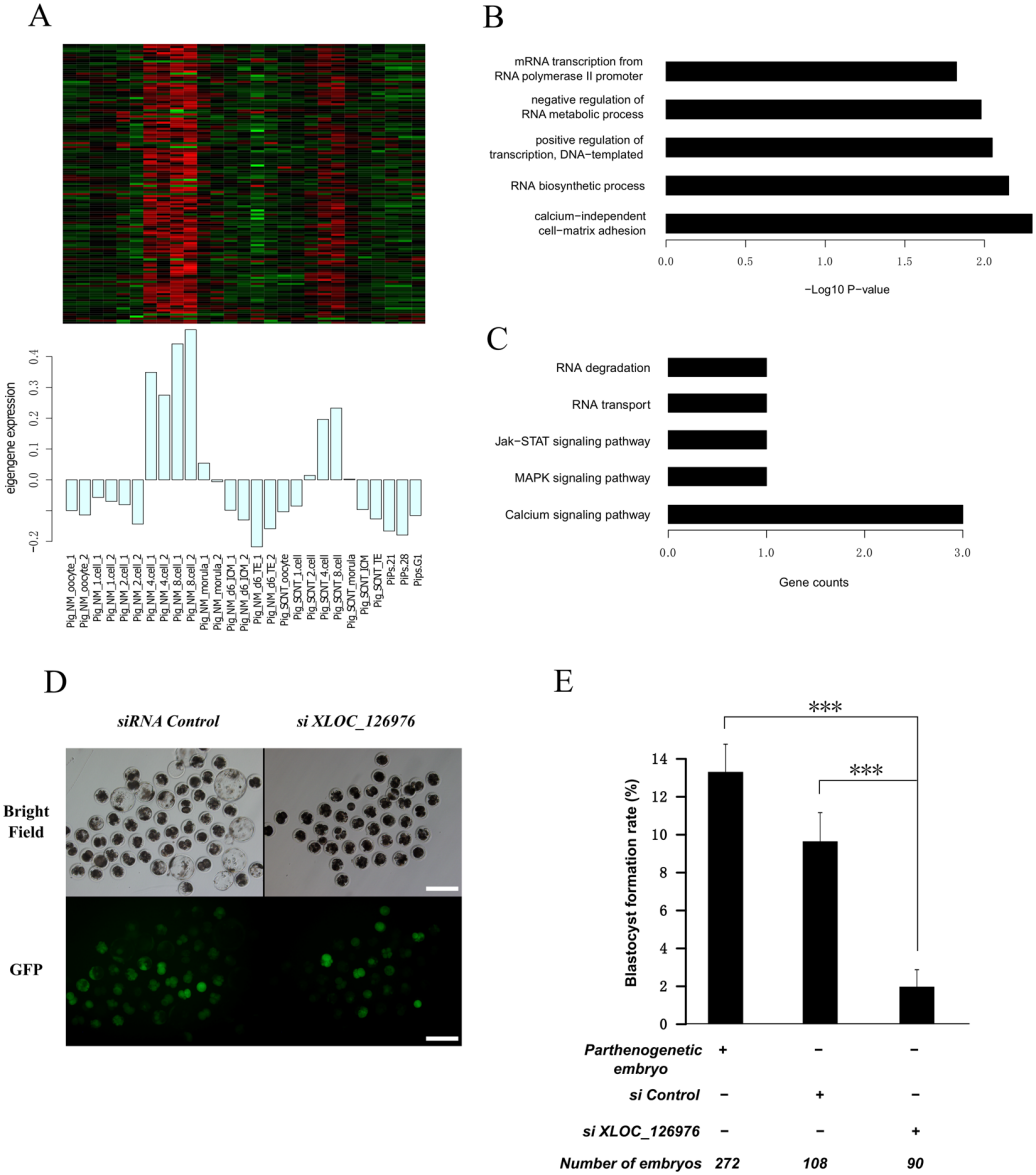
Co-expression network during ZGA stage. (**A**) Heatmap and bar plot of module lightcyan for the zygotic genome activation. The heatmap indicates the expression levels of genes in this module; all data were normalized with the z-score, and the bar plot indicates the module eigengene relative expression level. (**B**) Gene ontology enrichment for this module. (**C**) KEGG pathway enrichment for this module. (**D**) and (**E**) siRNA injection and the blastocyst formation rate. (**D**) The development state of the blastocyst after the siRNA of XLOC_126976 and the negative control were injected into parthenogenetically activated embryos. (**E**) The statistics for the blastocyst formation rate.

To verify whether the identified modules were biologically functional during the processes of pre-implantation embryonic development, we initially performed an enrichment analysis on the two stage using gene ontology (GO) and KEGG pathways. The lightcyan module was specifically and positively related in the two stages compared to the other modules that were significant in the 4-cell or 8-cell stage, although this module particularly showed a strong correlation and significance in the 8-cell (r = 0.71, *P* = 3 × 10^−5^) more than the 4-cell (r = 0.39, *P* = 0.04) stage (Fig. 4A). We identified 134 protein coding genes and 4 novel lncRNAs, which implies that lncRNAs may be involved in biological processes with protein coding genes. And we also search the neighborhoods of the 4 lncRNAs’ genomic locus, but unfortunately few genes were found and also independent with the co-expressed genes. Therefore we assumed the lncRNA worked in *trans* action. The lightcyan module was enriched for functional terms associated with the regulation of maternal product degradation, zygotic transcription and protein synthesis, including RNA biosynthetic processes (odds ratio = 2.17, *P* = 0.007) and positive regulation of transcription, DNA-template (odds ratio = 4.25, *P* = 0.009), and snRNA transcription (odds ratio = 100.9, *P* = 0.01). Furthermore, the comprised genes were enriched in analogous KEGG pathways, such as RNA degradation, RNA transport, ribosome biogenesis in eukaryotes and ubiquitin-mediated proteolysis. In contrast, the modules not related to the 4-cell or 8-cell stages had no significant enrichment in ZGA functions (see Supplementary Tables S5 and S6 for gene ontology and the pathway analysis associated with the darkred WGCNA module) (Fig. 4B).

Intriguingly, the most enriched functional term was the cellular response to lipoteichoic acid (odds ratio = 82.1, *P* = 5 × 10^−4^), which may play a role in the mediation of adherence and recognition process. Moreover, the second highest KEGG pathway enriched in this module was the calcium signaling pathway (odds ratio = 5.08, *P* = 0.028), which may be critical for the activation of zygotic genome transcription (Fig. 4C). The porcine pre-embryonic development was relatively controversial and confused; however, it corresponded to previous research. When the lncRNA that may regulate this process was knocked down, the embryos failed to further development to blastocysts, which was verified by the injection of siRNA into porcine embryos via parthenogenetic activation, particularly *XLOC_126976* in this module, which significantly reduced the blastocyst formation rate (Fig. 4D and Supplementary Fig. 7**)**. These findings suggested that the module was associated with maternal product degradation and ZGA; therefore, a regulatory network was constructed to illustrate the relationships among protein coding genes and lncRNAs with respect to these biologically relevant processes (Supplementary Fig. 8). We filtered and visualized the lncRNAs and protein coding genes for which the topological overlap of the network connections was greater than the default thresholds. Thus, the module demonstrates a clear relationship between lncRNAs and protein coding genes in the regulation of ZGA during the specific stage.

### The regulatory network of first lineage segregation during the blastocyst stage

**Figure 5:**
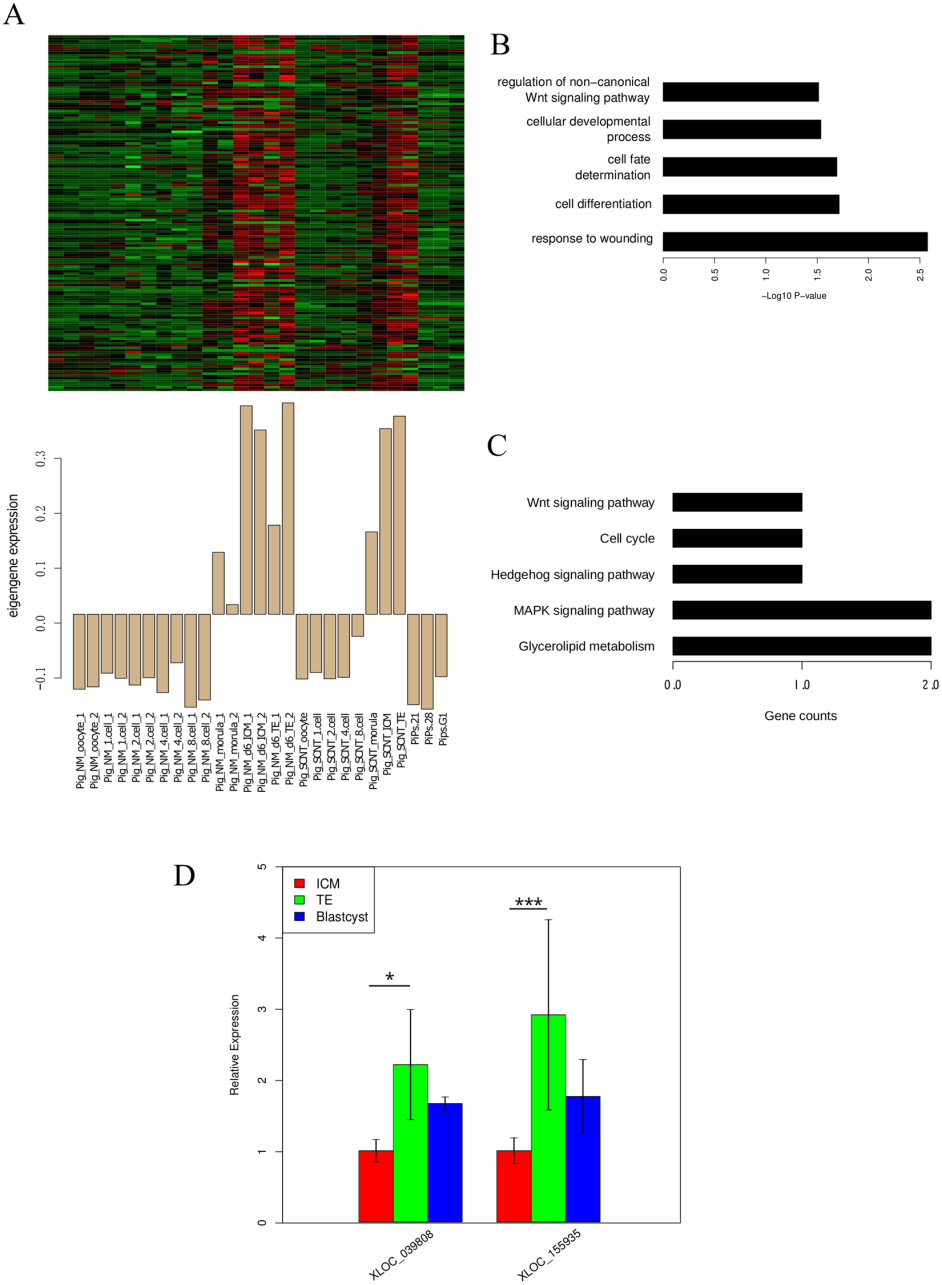
Co-expression network during first lineage segregation. (**A**) Heatmap and bar plot of module tan of the first lineage segregation. The heatmap indicates the expression level of genes in this module; all data were normalized with the z-score, and the bar plot indicates the module eigengene relative expression level. (**B**) Gene ontology enrichment for the module. (**C**) KEGG pathway enrichment for this module. (**D**) qPCR results of the 2 significant lncRNAs that were specifically expressed in the TE, *represent *P* < 0.05 and ***represent *P* < 0.01.

To investigate the molecular mechanism responsible for first lineage segregation, we selected the tan module (which was positively co-expressed during the morula to blastocyst stages) to perform the GO and pathway enrichment analyses, in this module ICM (r = 0.64, *P* = 3 × 10^−4^) was more significant than TE (r = 0.55, *P* = 0.003). In particular, compared to the morula stage, the tan module was more significantly associated with the blastocyst stage, which was separated into the ICM and TE (Fig. 5A). There were 151 protein coding genes and 2 lncRNAs in this module that were highly enriched for terms of biological processes related to cell differentiation (odds ratio = 3.04, *P* = 0.02), cell fate determination (odds ratio = 9.80, *P* = 0.02) and cellular developmental process (odds ratio = 42.89, *P* = 0.03) (Fig. 5B). Moreover, the hedgehog, MAPK and Wnt signaling pathways were also enriched in this module to mediate the lineage differentiation and choice. Interestingly, we determined that metabolism was significantly altered compared with other embryonic stages and was more different than the same components of the mouse blastocyst stage embryo, including the metabolism of fatty acids and glucose. In the module, the biosynthesis and metabolism of glycerolipid and glycosphingolipid were enriched, which may be accounted for more substantial volume of lipid droplets in the porcine embryo compared to the mouse embryo. These findings suggested that the potential function of this module may regulate the initial phase of the first cell fate determination in which two distinct lineages are formed in the blastocyst stage, namely, the ICM and TE **(**Fig. 5C and Supplementary Tables S7 and S8**)**.

The expression levels of *Cdx2* and *Nanog* were not significantly differentially expressed between the ICM and TE, probably because the structural and functional prediction is not well-established compared to humans and mice. Therefore, we attempted to take advantage of lncRNA as a potential marker to distinguish the ICM and TE. Furthermore, we determined that the two lncRNAs in the tan module were also specifically expressed in TE. Based on the qPCR results, we determined that *XLOC_155935* was significantly different (*P* = 0.007, Welch two-sample t-test) in the ICM, which was the same as the RNA-seq result (*P* = 0.02) (Fig. 5D and Supplementary Fig. 9). Moreover, supposing that *XLOC_155935* was a lineage-specific marker, we could have a better indicator of first lineage segregation, which generates the TE and ICM.

### Regulatory network of reprogramming to pluripotency

No validated porcine embryonic stem cell (ESC) lines have been established since the 1990s^30^; however, substantial efforts had been made to generate porcine pluripotent stem cells, such as putative pESCs and piPSCs. Porcine fibroblasts were used for reprogramming into iPSCs with four defined factors (*Oct4*, *Sox2*, *Klf4* and *c-Myc*). The piPSC colonies stained positive for alkaline phosphatase activity and had the ability to express surface markers of pluripotent stem cells (Supplementary Fig. 10). Moreover, compared to the porcine embryo data in the hierarchical cluster and PCA analyses, the piPSCs exhibited a high level of similarity to the porcine ICM that were used for the isolation of ES cell lines. These findings suggested that the piPSCs were successfully reprogrammed to pluripotency.

**Figure 6:**
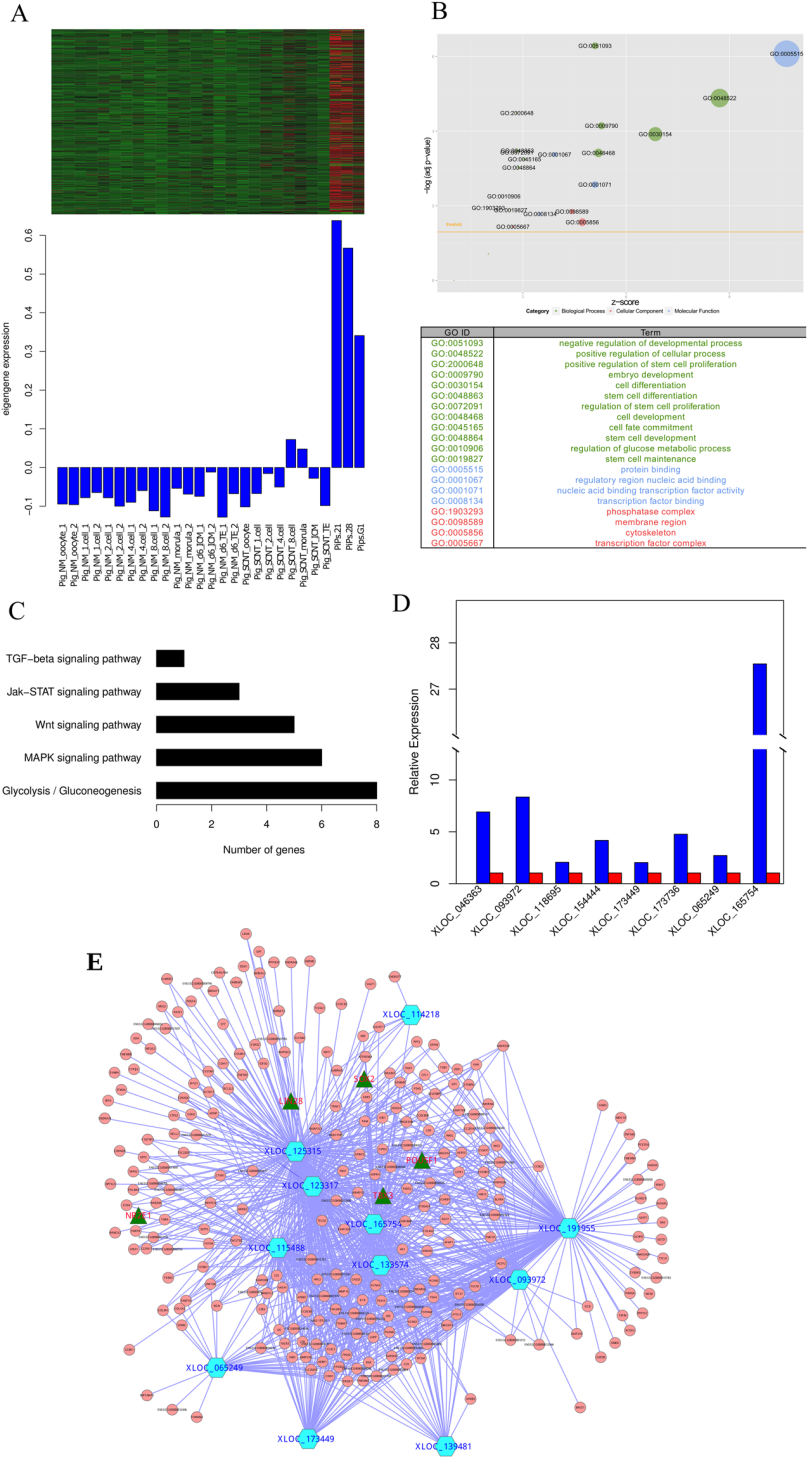
Co-expression network during reprogramming to pluripotency. (**A**) Heatmap and bar plot of module blue of the iPSC. The heatmap indicates the expression level of genes in this module; all data were normalized with the z-score method, and the bar plot indicates the module eigengene relative expression level. (**B**) Gene ontology enrichment for the module. (**C**) KEGG pathway enrichment for this module. (**D**) qPCR results for which the expression was significantly different between iPSC and pEF. (**E**) The lncRNA and protein coding gene regulatory network in iPSCs.

Furthermore, to investigate the potential roles of lncRNAs during the process of reprogramming, we performed a functional prediction enrichment analysis for the most significant module in iPS, the blue module, which contains 713 transcripts (702 protein coding genes and 11 lncRNAs) **(**Fig. 6A**)**. Genes in the blue module were enriched for functional terms associated with pluripotency, such as the positive regulation of stem cell proliferation (odds ratio = 15.83, *P* = 3.26 × 10^−5^), embryo development (odds ratio = 20.97, *P* = 7.17 × 10^−5^), stem cell differentiation (odds ratio = 2.43, *P* = 0.0003), stem cell development (odds ratio = 2.07, *P* = 0.001) and stem cell maintenance (odds ratio = 7.37, *P* = 0.011) (Fig. 6B). We also displayed a GOchord plot of the terms relevant to embryo development and stem cells between piPSCs and fibroblasts, which indicated that among the GO terms, the most up-regulated genes were over-represented in piPSCs (Supplementary Fig. 11). The pathway of this module was enriched in the Glycolysis/Gluconeogenesis, Wnt, MAPK, Jak-STAT, VEGF, p53 and TGF-beta signaling pathways (Fig. 6C). Energy metabolism shifted from oxidative phosphorylation to glycolysis with reprogramming to pluripotency and there is also a broad consensus of opinion regarding the Wnt, MAPK, Jak-STAT, and TGF-beta signaling pathways, which play critical roles in self-renewal, the maintenance of pluripotency and the identification of key molecules in the regulation of stem cell networks. We subsequently filtered the lncRNA and protein coding gene interactions based on the topological overlap matrix and constructed the piPSC-specific regulation network (Fig. 6E and Supplementary Tables S9 and S10).

To validate the expression levels of lncRNAs identified by the RNA sequencing in the piPSC-specific module, all lncRNAs in this module were selected for qPCR analysis. Compared to fibroblasts, the expression of *XLOC_165754* was the most significant lncRNA (*P* = 1.58 × 10^−8^, Welch two-sample t-test) and was not expressed in fibroblasts using RNA-seq (Fig. 6D**)**. We subsequently performed a synteny analysis to investigate whether *XLOC_165754* had co-localized and conserved blocks that linked two loci within two sets of chromosomes. *Cap2, Nup153, Kif13a* and *Dek* were identified as having shared synteny and were conserved genes ordered between the genomes of porcine and humans or mice with 5 kb upstream and downstream of the syntenic non-coding block (Supplementary Fig. 12**)**. These findings suggested that the iPSC module plays an important role in reprogramming to pluripotency, and *XLOC_165754* is the most likely lncRNA to indicate the pluripotent state in pigs through the promotion of reprogramming by the recruitment of transcription factors or epigenetic regulators.

### Epigenetic modification and conservation analysis for iPSC-specific lncRNAs

LncRNAs specifically expressed in different cell types indicated that epigenetic modifications may regulate the expression of lncRNAs by affecting their transcription. We determined that *XLOC_165754* was conserved across other species, such as mice and humans. The phastCons and phyloP scores were calculated for *XLOC_165754*. However, we determined that the conserved elements appeared to follow the coding exons reasonably well while also containing several noncoding regions, and the phyloP scores were fairly consistent with the phastCons scores. Intriguingly, the phyloP scores upstream of *XLOC_165754* were less than zero, which indicates a region of accelerated evolution (Supplementary Fig. 13). To investigate whether the lncRNA expression in iPSCs was activated or inactivated, we used previous ChIP-seq data (GSE36114) to validate our results. The broader peaks extended H3K4me3 at the TSS upstream of *XLOC_165754* and also had relatively broad peaks marked with the transcriptional regions of *XLOC_165754* (Supplementary Fig. 14), which suggested the character of *XLOC_165754* in accordance with previous studies^6^. H3K4me3 was bound up with the activation of lncRNA expression, whereas H3K27me3 was related to repression, which was responsible for the significantly differentiated expression between pEF and piPSCs. However, *Oct4*, *Sox2* and *Nanog* were not marked at the promoter regions of *XLOC_165754*; these three pluripotent transcription factors play important roles in the regulation of reprogramming and the maintenance of pluripotency for humans and mice^31^. These findings demonstrated that epigenetic modifications activated the expression of *XLOC_165754* to affect reprogramming and pluripotency, which may differ from the human or mouse core pluripotent networks and may involve other protein coding genes in porcine.

## Discussion

LncRNAs pose intriguing questions in developmental biology, particularly during pre-implantation embryonic development and somatic reprogramming. In this study, we systematically analyzed the lncRNA profiles of porcine pre-implantation embryos and iPSCs using RNA-seq data for the first time. To obtain high confidence lncRNAs, we filtered low-quality assemblies with an optimized FPKM threshold and identified 563 novel transcripts from 207 loci assigned low protein coding potential scores; however, the overall numbers of lncRNAs in this study is relatively small. We subsequently performed a weighted gene co-expression network and predicted the potentially functional regulatory network of stage-specific lncRNAs based on their associations with known protein coding genes involved in pre-implantation embryonic development and reprogramming to pluripotency. Moreover, we examined several potentially functional lncRNAs involved in embryonic developmental events and somatic reprogramming.

The novel lncRNAs in porcine embryos and iPSCs exhibit apparent similarities to other mammalian species. lncRNAs are typically shorter and have fewer exon numbers and lower expression levels than protein coding genes. Furthermore, lncRNAs exhibit more tissue-specific expression^1^ and less conserved sequence with other mammalian lncRNAs; however, some lcnRNAs have more positional conservation than sequence conservation across vertebrates^32^. Our findings also indicate these properties, which suggest that the identified lncRNAs are reliable. Due to different analytic methods, the genomic features of identified lncRNAs might show slight divergences. Compared to porcine lncRNAs in previous reports, we found that the lncRNAs in embryos and iPSCs (1157 nt) were shorter than those in endometrium (1454 nt) while longer than those in skeletal muscle (1043 nt). Besides, the number of exon in our result (3.3) was more than those in Wang and Zhao’s (~2.4) result^18,33^. The main reason is that a conservation method was applied in our identification pipeline, which leading to the highly conserved sequence of non-coding transcripts with human and mouse lncRNAs were chosen. Different analytic methods for identifying lncRNAs bring variation despite the genomic feature of lncRNAs in embryos and iPSCs in porcine was quite similar to former reports.

This study indicates modules of the regulatory network during embryonic development that are significantly enriched for genes involved in several developmental-specific stages. Due to the importance of embryogenesis, our data demonstrate 2 notable events during this period, including ZGA and first lineage segregation. In the ZGA period, genes were enriched for RNA biosynthesis and degradation. *Taf4* has been shown to mediate the RNA biosynthetic process and also to serve as a co-activator for developmental genes in Drosophila cells and vertebrates^34^. *Kdm3a* significantly altered the expression levels of maternal H3K9me2 and H3K9me3^35^, and at the transcriptional level, the H3K9 methylation patterns affect the switch of zygotic transcription^36^. Lipoteichoic acid mediates adherence and recognition between blastomeres, possibly due to the biases between blastomeres that result from an asymmetry of transcriptional trends during the 2- to 16-cell embryo stages^37^. Moreover, the impact of Ca^2+^ activated a stimulus to recruit specific maternal RNAs, which caused the activation of zygotic genome transcription^38,39^. Previous studies indicated that the 4-cell to 8-cell stage enriched lncRNAs, such as *XLOC_126976*, may play a role as a regulator that mediates the maternal to zygotic transition. The activation of these lncRNAs appears to be critical for the further development to blastocyst, which was consistent with siRNA injection with parthenogenetically activated embryos.

For the first lineage segregation, genes were enriched for cell fate determination and cell differentiation in this period. *Gata3* is notable in this module, which has been identified as a trophoblast factor and is co-expressed with *Cdx2* in blastocysts. *Gata3* and *Cdx2* may act in parallel pathways to induce the expression of the trophoblast lineage^40^. However, *Cdx2* was not significantly differentially expressed in blastocysts or co-expressed in this module. *Gata2* also plays a role in the determination of cell fate, and the expression level of *Gata2* is higher in the TE than the ICM in bovine blastocysts^41^. Thus, *XLOC_155935* may be regarded as a lineage-specific marker to TE instead of the absence of *Cdx2*. Furthermore, the metabolic characters of porcine pre-implantation embryos exhibit similarities and differences with other mammalian species. The similarities are represented in the well-established process of glycolysis^42^ and the increase in oxygen consumption during the blastocyst period^43^, oxidative phosphorylation was enriched in the blastocyst stage as expected because twice as much oxygen was consumed as in the cleavage stage embryos, which was consistent with previous research^30^; differences are present in fatty acid metabolism in which porcine embryos contain substantial amounts of lipid droplets, and the genes in this pathway are significantly upregulated, whereas this interesting phenomenon does not occur in mouse embryos^27^. These findings suggest that lncRNAs during porcine pre-implantation development are interesting given the dramatic transcriptional regularity that occurs in embryogenesis.

In addition, due to the similarity between iPSCs and ESCs derived from blastocyst inner cell masses in humans and mice^44^, we further investigated the lncRNAs and the regulatory network in piPSCs. Based on the principal component analysis and hierarchal clustering analyses, we determined the pattern of similarity between piPSCs and blastocysts, and the functional prediction of the genes in this module was also enriched for embryonic development. The Wnt signaling pathway could maintain the pluripotency in embryonic stem cells while playing a role in pre-implantation development and blastocyst lineage specification^45,46^. The inhibition of the MAPK signaling pathway could support the self-renewal of ESCs, whereas in mouse embryos, the MAPK signaling pathway promotes TE formation and blastocyst development^47,48^. LIF is a well-established cytokine for the maintenance of pluripotency and the self-renewal of mouse ES cells via the mediation of the Jak-stat signaling pathway, and SATA5 could also mediate the signals from cytokines that regulate pre-implantation development^49,50^. However, the VEGF and p53 signaling pathways could induce differentiation of embryonic stem cells in mice and humans, and the p53-p21 pathway served as a barrier in iPSC generation^51,52^. Furthermore, to our knowledge, piPSCs exhibit disparities in pluripotent states when cultured with different conditions^53^, and we not only remarkably determined that the surface markers were different but also showed that the iPSC stage-specific lncRNAs were differentially expressed. To examine the reprogramming associated lncRNA, we focused on the most significantly expressed lncRNA in iPSCs, *XLOC_165754*, which is expressed higher in hESC like piPSCs than mESC like piPSCs. Interestingly, with the synteny analysis, we showed that several counterparts up- and down-stream of *XLOC_165754* have a function in the maintenance of stem cell pluripotency^54,55^. Besides, according to the synteny analysis, we also found that the neighborhood of *XLOC_165754* such as *Nup153* could bind to the transcriptional start site (TSS) of developmental genes and mediate the recruitment of polycomb-repressive complex 1 (PRC1) to maintain stem cell pluripotency by functioning in mammalian epigenetic gene silencing^54^; and in a comparison of the differentiation into cardiac precursors, *Kif13a* is the most highly expressed in the hESC lines^55^. However, no authentic porcine ESCs have been derived, whereas piPSCs will not remain stable without exogenous genes. The co-expression network suggests that with the exception of *Oct4* and *Sox2*, endogenous *Lin28* and *Tbx3* are likely to be crucially involved in the pluripotent network, which could provide clues for the generation of high quality piPSCs.

Many studies have been conducted in mice^56^, humans^57^ and other model organisms^10^ to explore how embryonic development and reprogramming to pluripotency are regulated by lncRNAs and mRNAs; however, to date, the mechanism reported with respect to porcine pre-implantation embryos and pluripotent stem cells has been extremely limited. RNA-seq is becoming an attractive approach to profile gene expression levels with respect to decreasing sequencing costs. However, RNA-seq is confined by technical limitations that make it difficult to profile mRNA and lncRNA transcripts in porcine pre-implantation embryos due to the small number of embryonic cells and the identification and analysis of high confidence lncRNAs at relatively lower expression levels. Moreover, a blastocyst that is separated into the ICM and TE via a surgical procedure may result in a cellular response to the damage, which may be responsible for genes enriched with functions in immune or inflammatory responses. Our study identified novel lncRNA candidates; however, the accuracy could be further improved via stand-specific deep sequencing. Moreover, for porcine embryos, it is important to utilize the single cell sequencing technologies capable of providing more unbiased methods to profile the transcriptomes.

In conclusion, we identified high confidence lncRNAs involved in the processes of pre-implantation development and somatic reprogramming. Notable co-expressed modules were identified in several important events during these periods; thus, we predict the potentially functional lncRNAs and their regulatory networks with protein coding genes. Our study indicates that the transcriptional regularities of lncRNAs during embryonic development and reprogramming to pluripotency are highly dynamic and stage-specific, which will enable further investigation of these lncRNAs and may provide clues for the generation of high quality pluripotent stem cells as well as the derivation of embryonic stem cells from porcine blastocysts.

### Accession Numbers

Raw reads of porcine embryos and iPSC RNA-seq data has been submitted to the NCBI Sequence Read Archive (SRA; http://www.ncbi.nlm.nih.gov/sra/) under accession number SRA326708.

## Methods

### Biological materials

Young adult female C57 mice (Vital River Laboratories, Beijing, China) and young adult female Nong Da Xiang mini-pigs (China Agricultural University pig farm, Zhuo Zhou, China) were kept at 25 °C, fed *ad libitum* and a 24 h light-dark cycle (12 L:12D). All animal procedures and all of the animal works were approved by the Animal Care and Use Committee of China Agriculture University (Permit Number: SKLAB-2016-05-01). All methods were carried out in accordance with relevant regulations and guidelines of State Key Laboratory for Agrobiotechnology, China Agricultural University.

### Embryo collection and RNA isolation

Oocytes were collected via superovulation by an initial intraperitoneal injection of 5 IU Pregnant Mare’s Serum Gonadotropin (PMSG) followed 48 h later by 5 IU human chorionic gonadotrophin (hCG). At 14 h after the last intraperitoneal in injection oocytes were flushed from the oviducts. The *in vivo* pig embryos were washed from the oviduct or uterus with phosphate-buffered saline (PBS) containing 5% fetal bovine serum (FBS) after estrus and natural mating. We use the Nong Da Xiang mini-pigs to obtain the oocytes and fibroblasts that respectively refer to the gametes and the donors in somatic cell nuclear transfer. Then through oocyte maturation, cell cycle synchronization of donor cells, enucleation, cell fusion, oocyte activation and embryo culture, we collected the *in vitro* embryos. The blastocyte segregated TE and ICM physically by an ultra-sharp splitting blade (Bioniche, Animal Health US, Inc) with a stereomicroscope.

After the collection of embryos, we mixed 4–10 embryos at the same stage together for each sample and transferred and centrifuged into a 0.5 mL tube with 50 μL extraction buffer from the PicoPure RNA Isolation Kit (Arcturus, KIT0204, Life Technologies, US) at 42 °C for 30 min. The extract was then held at −80 °C until RNA isolation. Total RNA was isolated from each sample according to the manufacturer’s protocol and eluted into 10 μL elution buffer.

### RNA sequencing

The total RNA was used as starting material to select poly-A RNA and used for constructing SOLiD libraries according to the protocols supplied by the Applied Biosystems SOLiD 4 System manufacturer. RNA samples were spiked in with NIST standards before libraries were constructed. RNA isolated from each embryonic sample was used for double-stranded cDNA synthesis, PCR amplification. The fragment library was prepared according to the Library Preparation Protocol for whole transcriptome analysis of a single cell and the Applied Biosystems SOLiD 4 System Library Preparation Guide (http://www.appliedbiosystems.com). The RNA-seq libraries were sequenced on ABI SOLiD sequencing platform as 50-base reads according to the manufacturer’s recommendations.

### Reads mapping and transcripts assembly

Short reads were mapped to the mm9 (mouse) and susScr2 (pig) reference genome with Tophat2^58,59^. The annotation files were obtained from the Ensembl database: mouse (NCBIM37.65) and pig (Sus scrofa9.65). Aligned reads of each sample were assembled with cufflinks (version 2.21)^60^ and we performed Reference Annotation Based Transcript (RABT) assembly^61^ with the Ensembl annotation to compensate incompletely assembled transcripts caused by read coverage gaps in the regions of known genes in the assembly. We merged together all cufflinks assemblies and used cuffcompare to filter the transcripts with those annotations in Ensemble gene, Refseq gene and UCSC gene. Then we screened the transcripts with those transfrag class codes: ‘i’ (A transfrag falling entirely within a reference intron), ‘j’ (Potentially novel isoform or fragment that at least on splice junction is shared with a reference transcript), ‘o’ (generic exonic overlap with a reference transcript), ‘u’ (intergenic transcript) and ‘x’ (exonic overlap with reference on the opposite stand).

### Long non-coding RNA Identification

We first filter the high confidence transcript with a cutoff defined in Supplementary Fig. 1. Then we extracted the transfrags class code marked with ‘i’ (a transfrag falling entirely within a reference intron), ‘j’ (potentially novel isoform), ‘o’ (generic exonic overlap with a reference transcript), ‘u’ (intergenic transcript), and ‘x’ (Exonic overlap with reference on the opposite strand) which may be lncRNA candidates and subsequently excluded the single exon, short exonic length (<200 nt) and short open reading frame (<300 nt) transcripts. We performed both the Coding Potential Calculator (CPC) and Coding-Potential Assessment Tool (CPAT) to calculate the coding potential, as well as PhyloCSF to analyze the conserved regions of the multi-species genome sequence alignment of all candidate transcripts to distinguish the coding and non-coding sequences. Furthermore, the transcripts were searched against the Pfam database of hidden Markov models (HMMs) to filter the sequences that contained protein domains.

### Weighted Gene Co-expression analysis Network Analysis (WGCNA)

Although method of WGCNA was designed for the analysis of microarray data, it has been already applied to RNA-Seq data^62,63^. Gene expression FPKM values were log2 (x + 1) transformed before being processed through the WGCNA R package^29^. We applied co-expression analysis to identify the specific modules of highly correlated genes and lncRNAs by the referenced methods^64^ and an unsigned network was constructed via creating a matrix of pairwise correlation between all pairs of genes across the samples. Based on the scale free topology, we picked up the most proper soft-thresholding power^65^. The max block size was set to 12000 and the minimum module size was set to 75. Dynamic hybrid tree cut algorithm was used to cut the hierarchal clustering tree, and the branches from the tree cutting were used to define modules. Eigengenes whose correlation value greater than 0.75 in each module were merged and the first principal component of each individual module was used to evaluate their relationship with cell type. Other parameters were used with the default to construct a block-wise network.

### Differential expression analysis and function enrichment analysis

For protein coding genes, we use HTseq^66^ to obtain read count tables from binary sequence alignment/map (BAM) file. The DEseq 2^67^ package was used to normalize count data and calculate the differential expression tests between each two groups associated cell type. A gene or lncRNA will be reported significant differential expression gene if the DESeq 2 results show that the p-value was less than 0.05 and the absolute log2 fold change was greater than 1. The Gene Ontology (GO) functional and KEGG pathway enrichment analyses of differential expression genes and WGCNA module genes were performed in R/Bioconductor using the package: GOstats, GSEA^68^ to compute the statistics and GOplot^69^ to visualize the function analysis. All the statistical significance described above was p-value.

### Cell culture

The porcine embryonic fibroblasts (pEFs) were isolated from embryos (approximately embryonic 26-30d) arising from Nong Da Xiang. pEFs were cultured in DMEM medium containing 10% fetal bovine serum (FBS), 1% non-essential amino acid, 1% GlutaMAX-L and 1% penicillin/streptomycin (Gibico). 293-GP2 cells were cultured in DMEM with 10% FBS that used for viral packaging. The generated piPSCs were cultured in the commercial medium (NutriStem XF/FF Culture Medium, Stemgent 01-0005), and were incubated at 38 °C at 5% CO_2_. The piPSCs were passaged by 0.1% collagenase IV (Gibico).

### The generation of piPSCs

To perform the reprogramming of pEFs, 4 retroviral vectors were constructed with porcine *Oct4*, *Sox2*, *c-Myc* and *Klf4* respectively. Retroviral vectors production was conducted according to the method reported previously^70^. pEFs were infected with the indicated combinations of retroviruses for 12 h with 8 μg/ml polybrene (Sigma, 107689). The infection was performed twice. Infected pEFs were passaged and seeded into the plated feeder layer, which was inactivated mouse embryonic fibroblast (MEF) with mitomycin C. Then changing the pEF medium to piPSC medium on the second day and the medium was changed every two days. The reprogramming efficiency was evaluated by AP staining at day 15. Colonies were picked up at day 30.

### Quantitative real-time PCR analysis

Total RNA of piPSCs and porcine fibroblast were extracted with an RNeasy Mini Kit (QIAGEN, 74104). The reverse transcription was conducted with Oligo-dT primer and M-MLV Reverse Transcriptase (Promega, M1701). Quantitative real-time PCR was conducted with LightCycler 480 SYBR Green I Master Kit (Roche, 4887352001) according to the manufacturer’s instructions, Ct (fit point method) and Cp (second derivative method) values were detected with LightCycler 480II (Roche) and exported into an MS Excel data sheet (Microsoft) for analysis after background subtraction. The data was analyzed with the comparative Ct (2^−ΔΔCt^) method. The ΔCt was calculated using EF1-α as a internal control. All experiments encompassed at least 3 biological replicates. The primers used in these analyses are listed in Supplementary Table S1.

### Conservation analysis

PhastCons scores and PhyloP scores were used to estimate the conversation level of long non-coding transcripts. We download the pairwise alignments of susScr2 among human (hg19), mouse (mm9) and cow (bosTau7) that is best chained or with gaps in the best chains filled by the next-best chains where possible in the susScr2 genome. Then group blocks of chained alignments into longer stretches of synteny and recreate the multiple alignments. We conduct PhastCons^71^ to calculate the whole genome phyloP score and phastCon score with default parameters. The phyloP and phastCons score of long non-coding transcripts were visualized by RPHAST^72^.

### ChIP-seq analysis

To identify the porcine lncRNAs whether involved in pluripotency in piPSCs, we use a series of histone modification and DNA-binding factors datasets in GSE36114 which was downloaded from Gene Expression Omnibus (GEO). After quality check (QC) we mapped the raw reads with bowtie against susScr2. MACS2^73^ were used to process for the peak calling. We integrated the RNA-seq data and ChIP-seq data and visualized them in Integrative Genomisc Viewer (IGV).

### siRNA injection of porcine embryos by parthenogenetic activation

We microinjected 5–10 pl of 2 µM siRNA that targeted lncRNA that regulated zygotic genome activation, together with GFP mRNA, into the parthenogenetically activated embryos. All the siRNAs that targeted ZGA associated lncRNA are listed in Supplementary Table S2.

## Electronic supplementary material

Supplemental_Figures 1-14

Supplementary_Table 1-11
