# Supplementary material for: Long non-coding RNAs involved in the regulatory network during porcine pre-implantation embryonic development and iPSC induction
Source: Sci Rep. 2018 Apr 27;8:6649. doi: 10.1038/s41598-018-24863-5 (PMC5923264; doi:10.1038/s41598-018-24863-5)
Supplement: Supplementary file 1 — Supplemental_Figures 1-14 [file 41598_2018_24863_MOESM1_ESM.pdf]

Long non-coding RNAs involved in the regulatory network during porcine pre-implantation embryonic development and iPSC induction

Zhong Liang[1,2], Mu Haiyuan[2], Wen Bingqiang[2], Zhang Wei[2], Wei Qingqing[2], Gao Ge [3], Han Jianyong[2,*] and Cao Suying[1,4,*]

[1] The Animal Science and Technology College, Beijing University of Agriculture, Beijing, China

[2] State Key Laboratory for Agrobiotechnology, College of Biological Sciences, China Agricultural University, Beijing, China

[3] State Key Laboratory of Protein and Plant Gene Research, College of Life Sciences, Center for Bioinformatics, Peking University, Beijing, China

[4] Beijing Key Laboratory of Traditional Chinese Veterinary Medicine, Beijing University of Agriculture, Beijing, China

Tel: 8601062734378;

Fax: 8601062734378;

*Correspondence: Cao Suying, Han Jianyong

Email: 20137602@bua.edu.cn; hanjy@cau.edu.cn

SUPPLEMENTARY FIGURE 1 – The distribution of RNA-seq expression

Black, density plot of log-transformed FPKM values of protein coding genes. Red, Gaussian fit to the log-transformed FPKM values. The top of the Gaussian fit distribution of FPKM treat as the threshold for high confidence transcript. The minimum log2 FPKM value was defined as -15, μ is the FPKM at the kernel density estimation maximum, U is the mean of all log2(FPKM),the Gaussian fit distribution list below:

$$\sigma = (U - \mu)\sqrt{\frac{\pi}{2}} \quad (1)$$

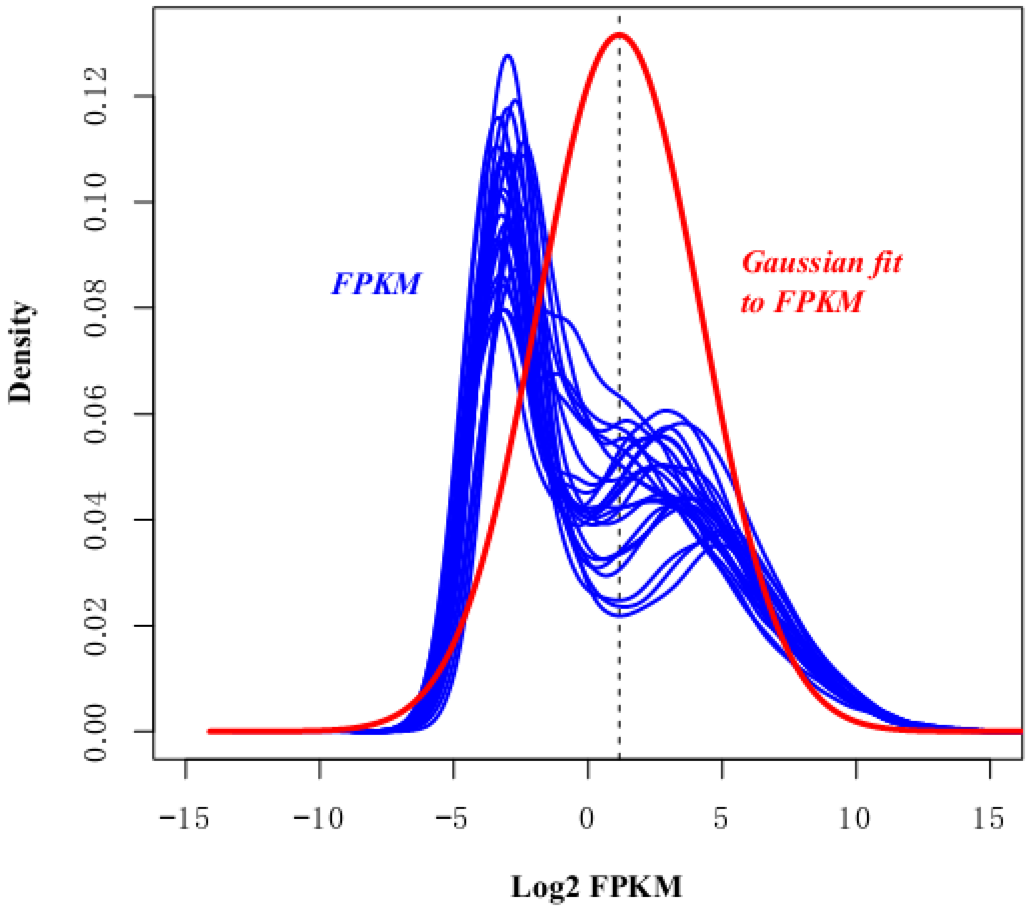

SUPPLEMENTARY FIGURE 2 – Global profiling of the porcine pre-implantation embryo and iPSC transcriptome

Expression that incorporated protein coding genes and lncRNAs, FPKM was transformed with log10. The most outside of the circle represents the number of chromosome and the length of outer circle shows the size of chromosome. The whole genome is divided according to the scale of 1 Mb. If there are 2 or more genes and long non-coding RNAs within 1 Mb, the expression is calculated according to the sum of expression level of all transcripts. The histograms from the outside to the inside represent that the expression level (FPKM) of oocytes, 1 cell, 2 cell, 4 cell, 8 cell, morula, inner cell mass, trophectoderm and iPSC cells in turn. Red histograms indicate that the expression level of predicted long non-coding RNA.

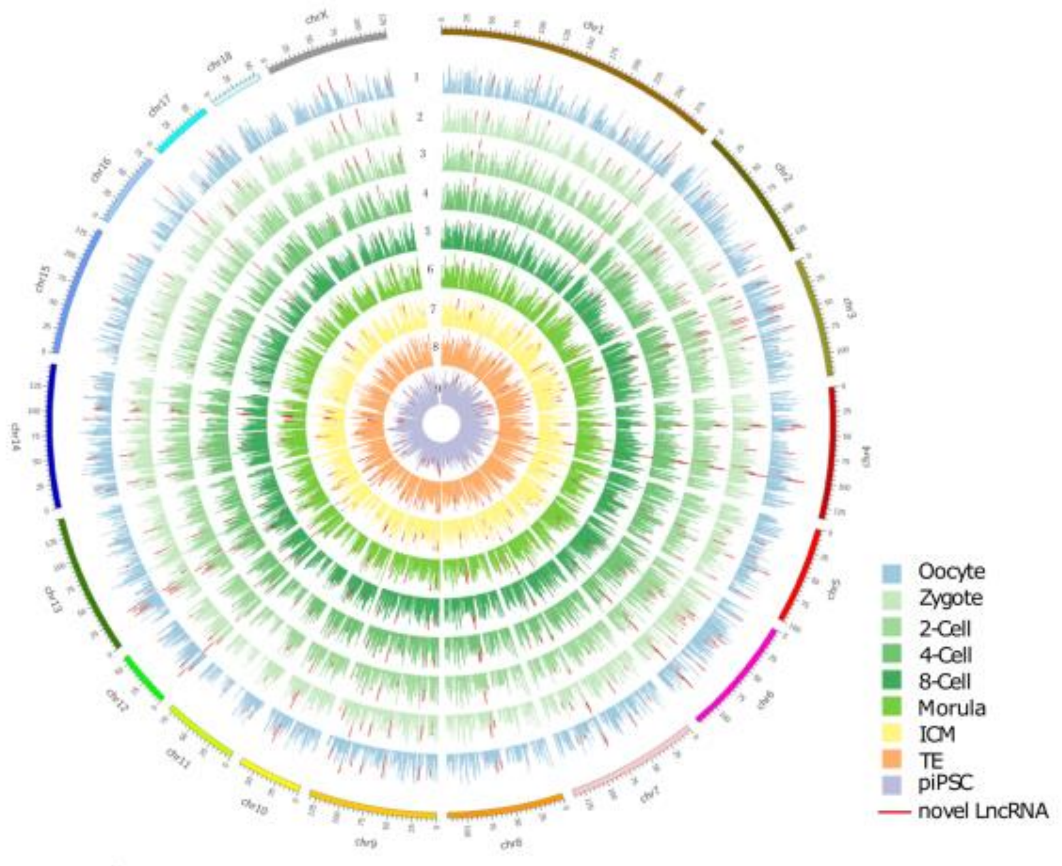

SUPPLEMENTARY FIGURE 3 – Hierarchical clustering analysis for porcine iPSCs and blastocyst

Hierarchical cluster analysis of both protein coding genes in iPSCs and blastocyst, ICM and TE were separated as mentioned in methods. We chose the genes associated to pluripotent and embryonic development and the data was transformed with log 2. The agglomeration method used is WPGMA (Weighted Pair Group Method with Averaging). AU (Approximately Unbiased) in red color represents p-value and BP (Bootstrap Probability) value in green color. AU p-value is computed by multiscale bootstrap resampling method

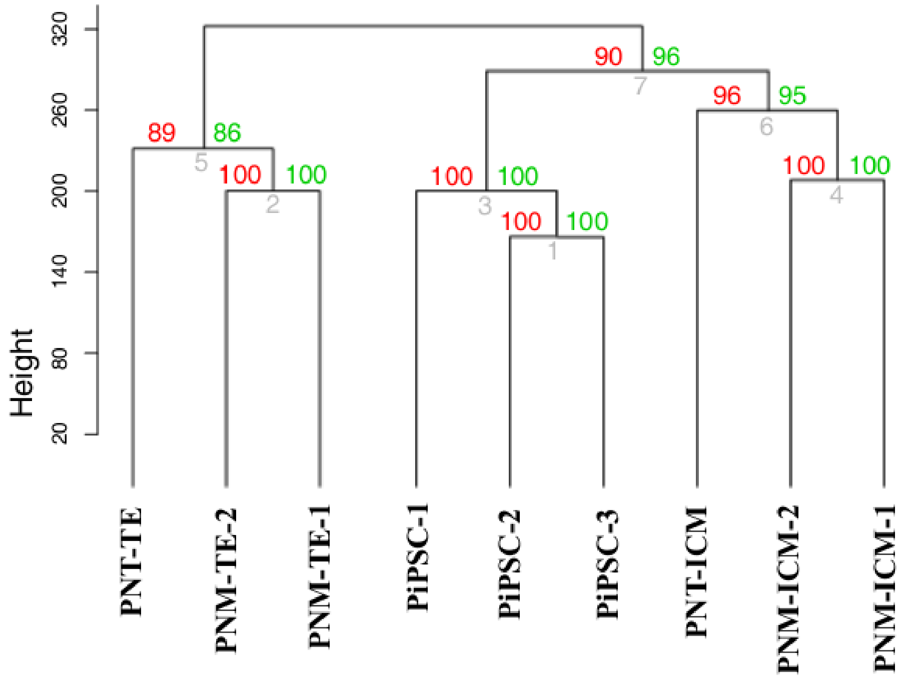

SUPPLEMENTARY FIGURE 4 – Pick optimized parameters for the co-expression network

The left side the figure show the soft threshold of the network, we based on the criterion of the approximate scale-free topology to select the most proper soft threshold power = 9, the red line on the square means the $r^2 > 0.9$ in the model. The right side show the mean connectivity under the soft threshold.

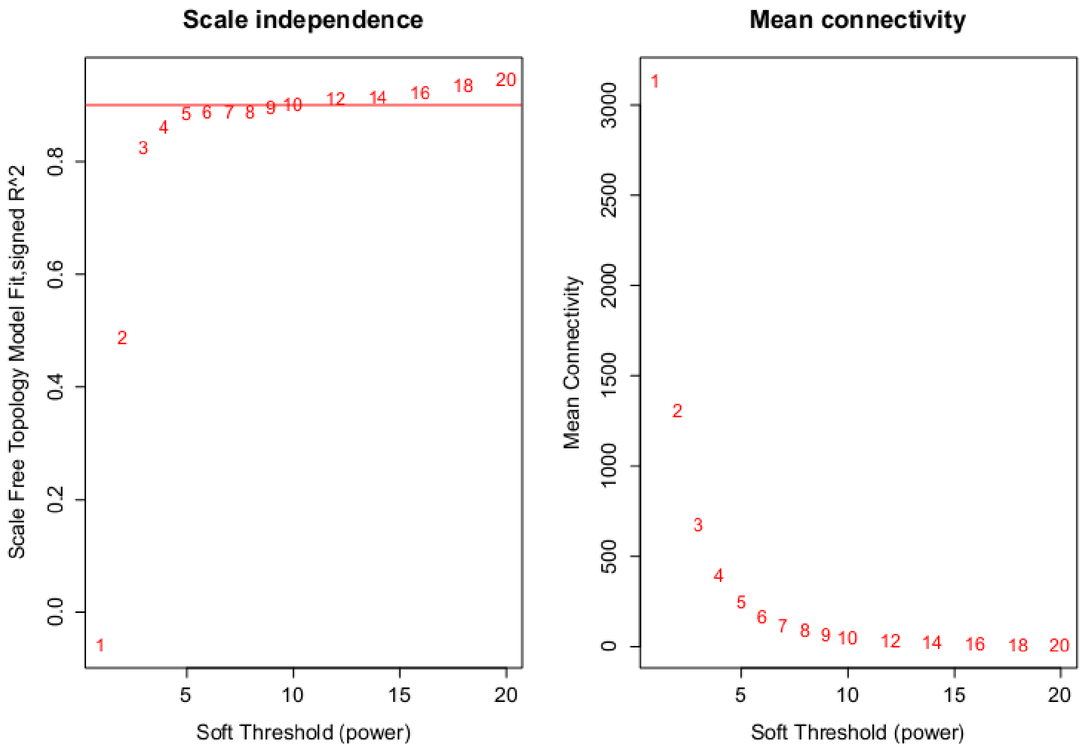

SUPPLEMENTARY FIGURE 5 – Gene dendrogram and module colors in pig RNA-seq

Dynamic Hybrid Tree Cut algorithm was used to cut the hierarchal clustering tree and defined modules as branches from the tree cutting, eigengenes whose correlation value greater than 0.75 in each module were merged and the first principal component of each individual module was used to evaluate their relationship.

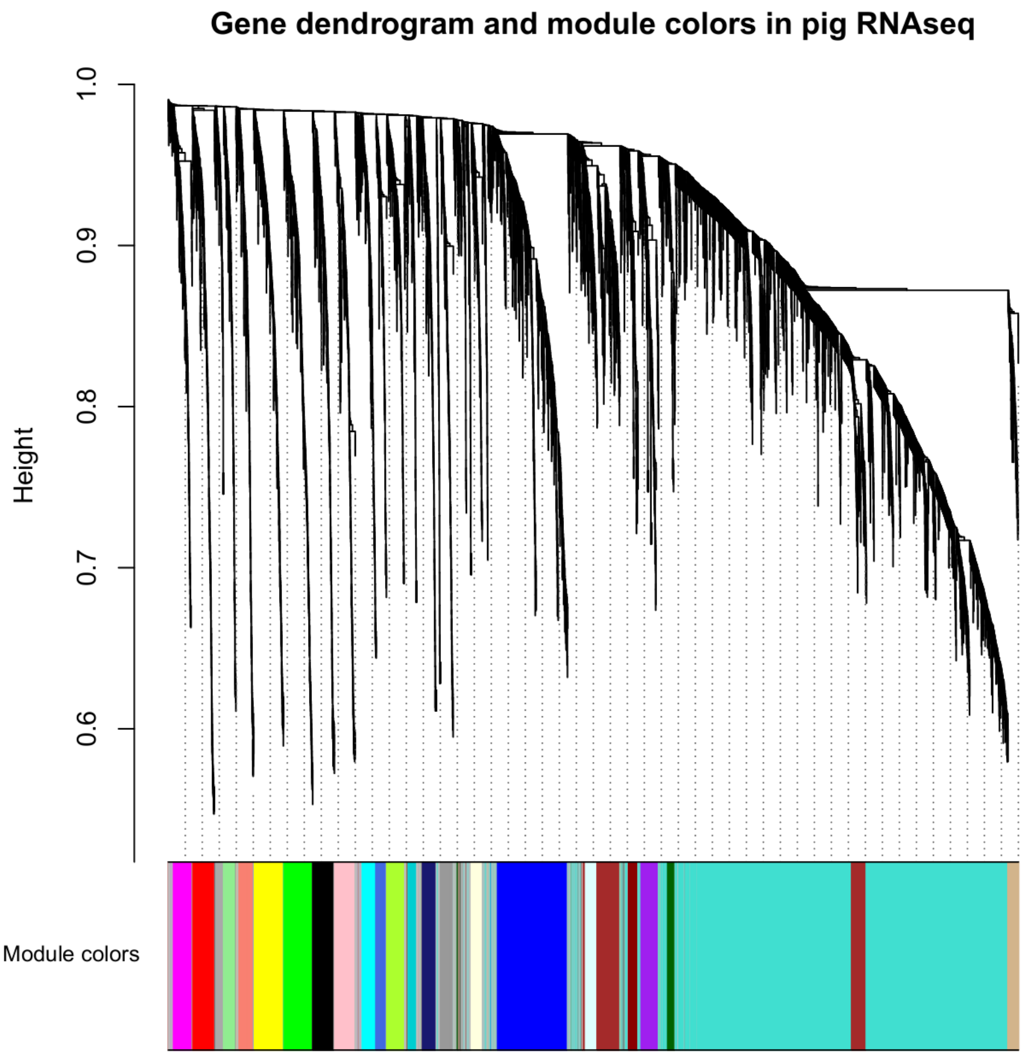

SUPPLEMENTARY FIGURE 6 – Stage-specific functional modules prediction of lncRNAs and protein coding genes

Heatmap showing the significance of modules between independently constructed gene set and cell type. The x axis shows only cell type (n = 9) and the y axis shows all signifcant modules (n = 25). Each cell contains the correlation and P-value of the mudule.

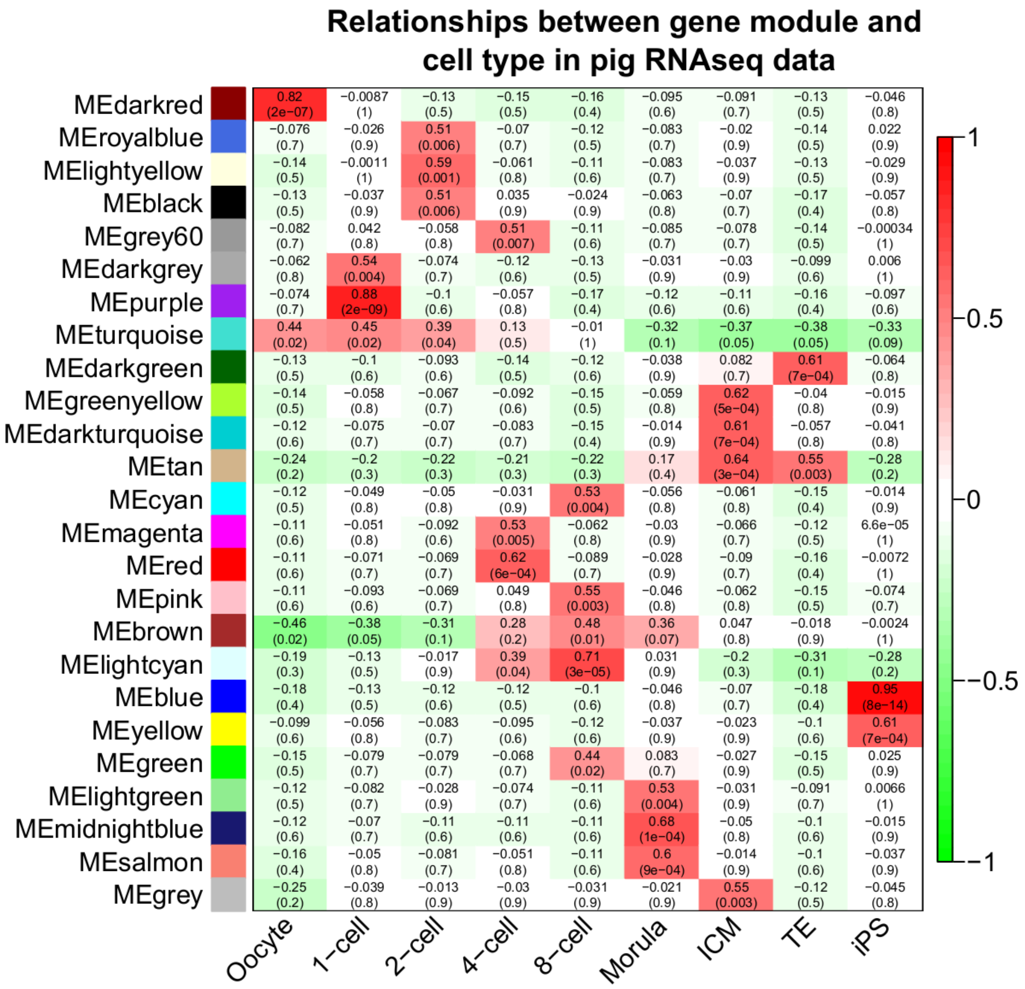

SUPPLEMENTARY FIGURE 7 – Effect of lipofection of siRNA after 24 h in piPSC

We perform knockdown experiment of *XLOC_126976* in piPSC with siRNA. siRNA include XLOC_126976-1 and XLOC_126976-2 which was lipofected into piPSC and cells were collected after lipofecting 24 h. The expression of *XLOC_126976* in negative control significant higher than those infected with siRNA. * $P < 0.05$.

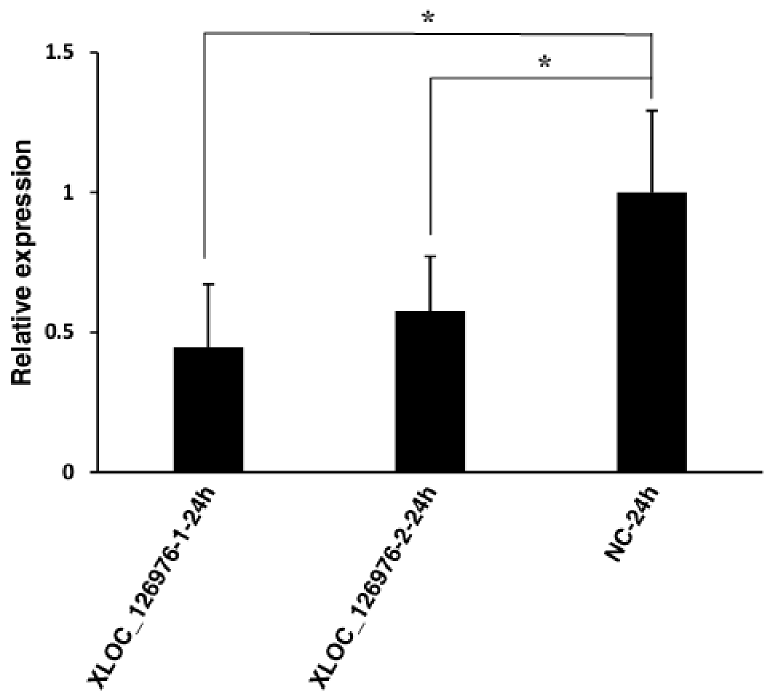

SUPPLEMENTARY FIGURE 8 – Co-expression network between lncRNA and coding genes of module lightcyan

lncRNAs and protein coding genes co-expression network of ZGA module. The hex shape is the lncRNA and the circle shape is the protein genes.

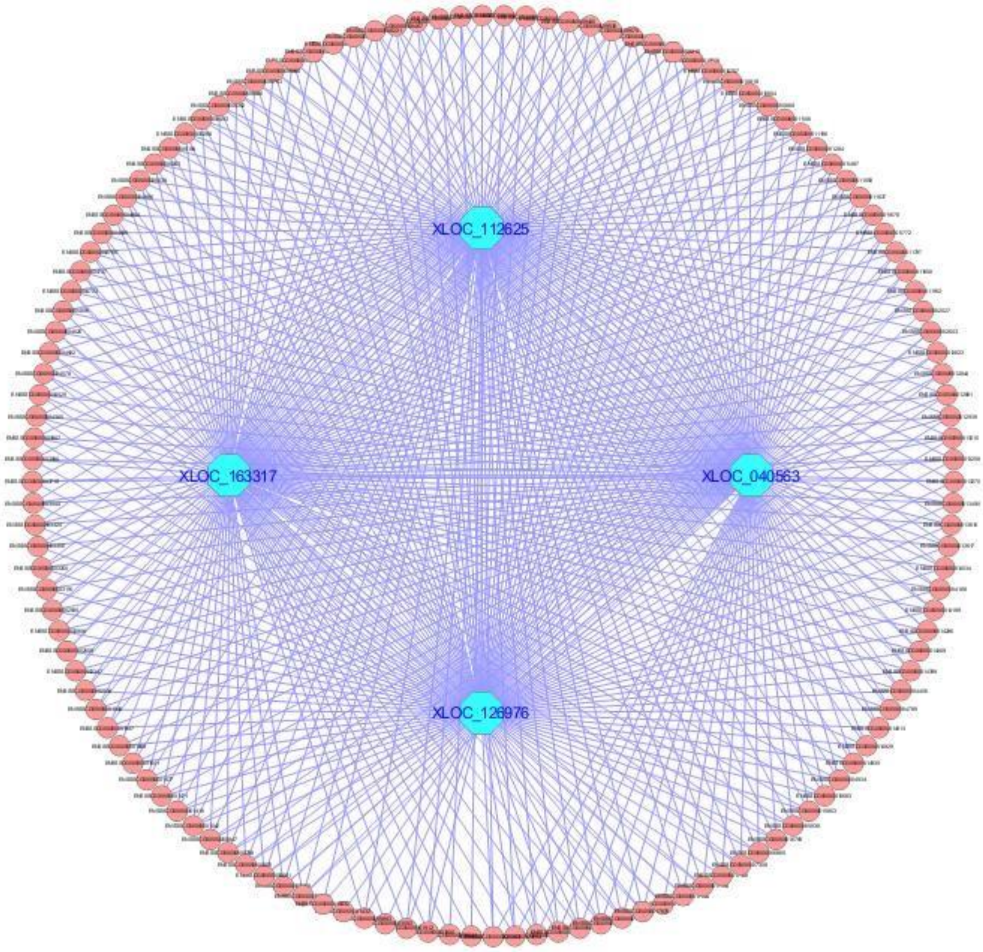

SUPPLEMENTARY FIGURE 9 – RNA-seq result of XLOC_155935

The expression level of *XLOC_155935* in each stage of embryonic development and reprogramming, which was specifically expressed in blastocyst. *XLOC_155935* was identified as potential marker for TE, which significantly up-regulated in TE than ICM (P = 0.02).

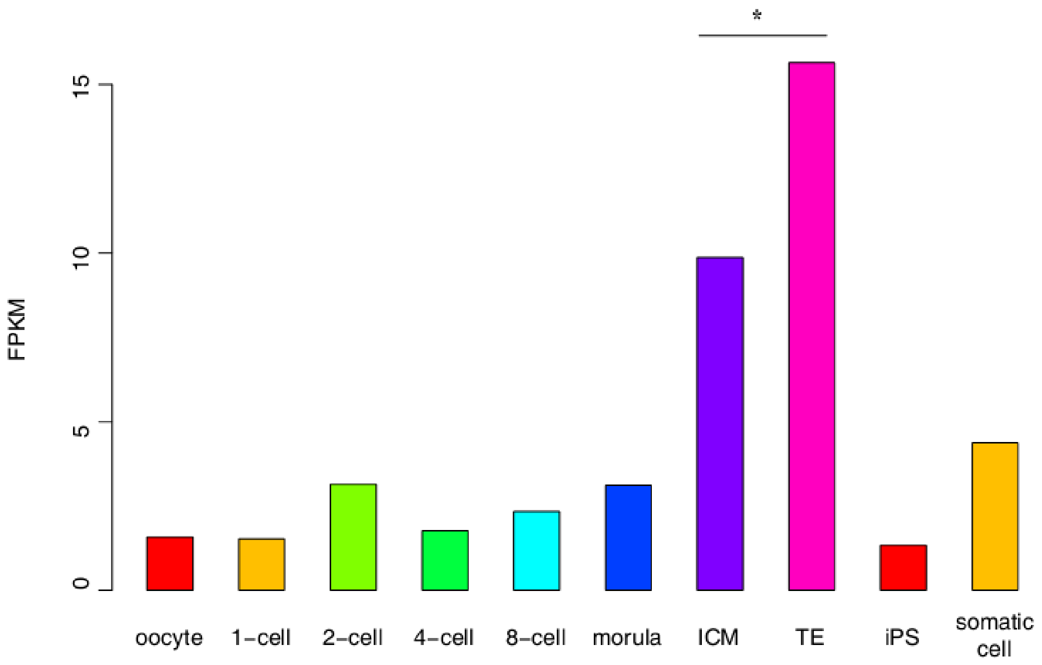

SUPPLEMENTARY FIGURE 10 – AP and Immunocytochemical staining of piPSCs

(A) The left side is colony of iPSC in the bright field and the right side is alkaline phosphatase (AP) staining for iPSC. Bar scale = 200 μm. (B) The Immunocytochemical staining of pluripotent and surface marker of piPSC: Oct4, Sox2, Tra-1-81 and Tra-1-60. Bar scale = 200 μm.

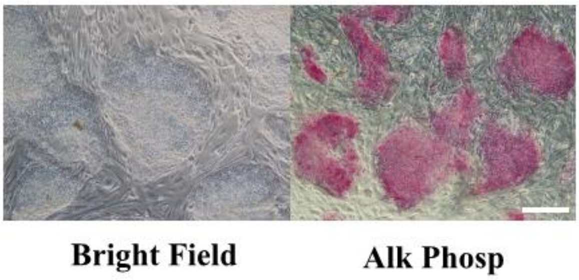

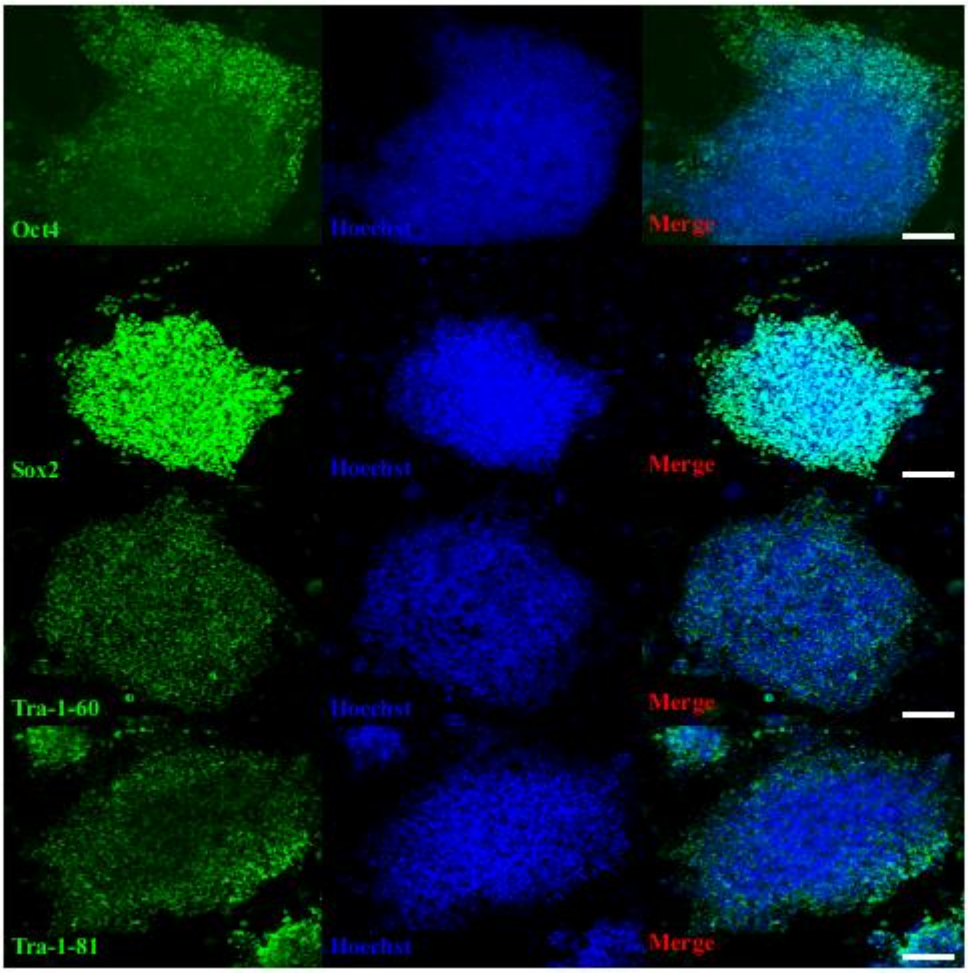

SUPPLEMENTARY FIGURE 11 – Genes of MEblue play a important role in regulating embryonic development and stem cell self-renewal

Genes in this module combined Log2 fold change and enriched functional term of embryonic development and stem cell self-renewal to draw a Circos plot. The left outer ring is gene name and the red and blue blocks in close proximity to gene name is Log2 fold change of iPSC cells versus fibroblasts, the color blocks in the right outer ring represent the functional term of GO, the colored linkages inside the ring represents of the related genes under the function with specific color.

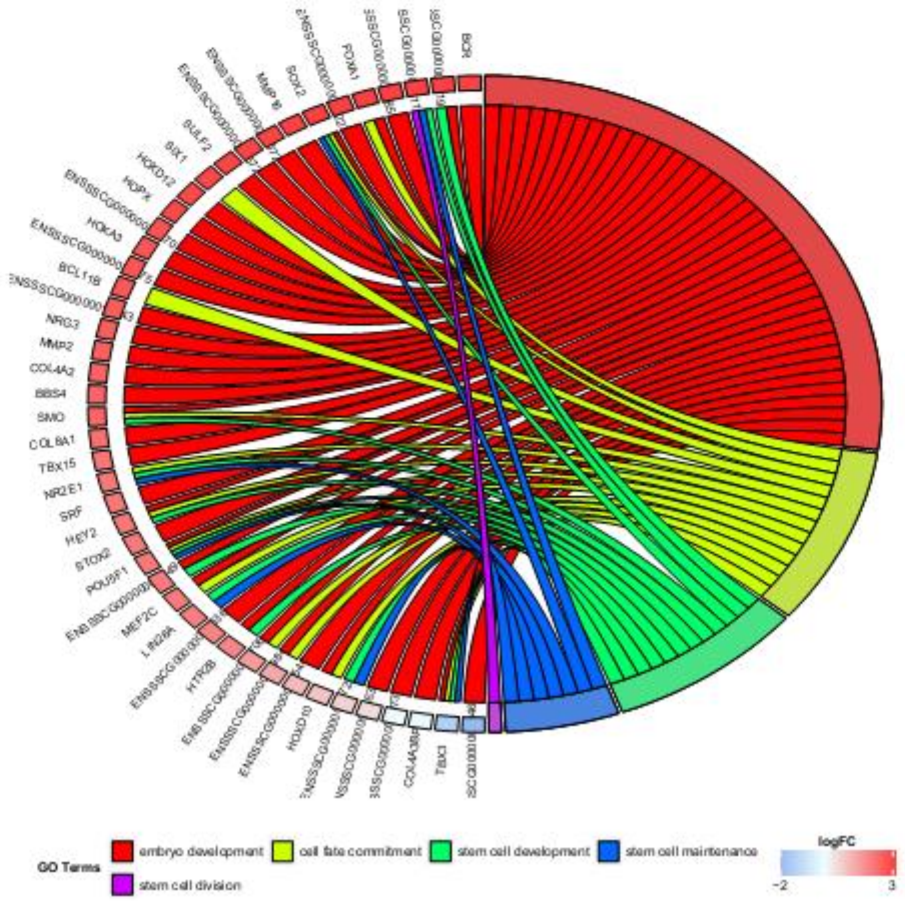

SUPPLEMENTARY FIGURE 12 – Synteny analysis for *XLOC_165754*

The counterpart of XLOC_165754 (chr7:14452777-14481617, displayed with a black arrow) in human and mouse genome. The block in grey represent the protein coding genes and the block in red represent the non-coding RNA. Pig genome in the middle, mouse genome in the left and human genome in the right. The grey lines between two genomes represent the same genes in each counterpart of genome.

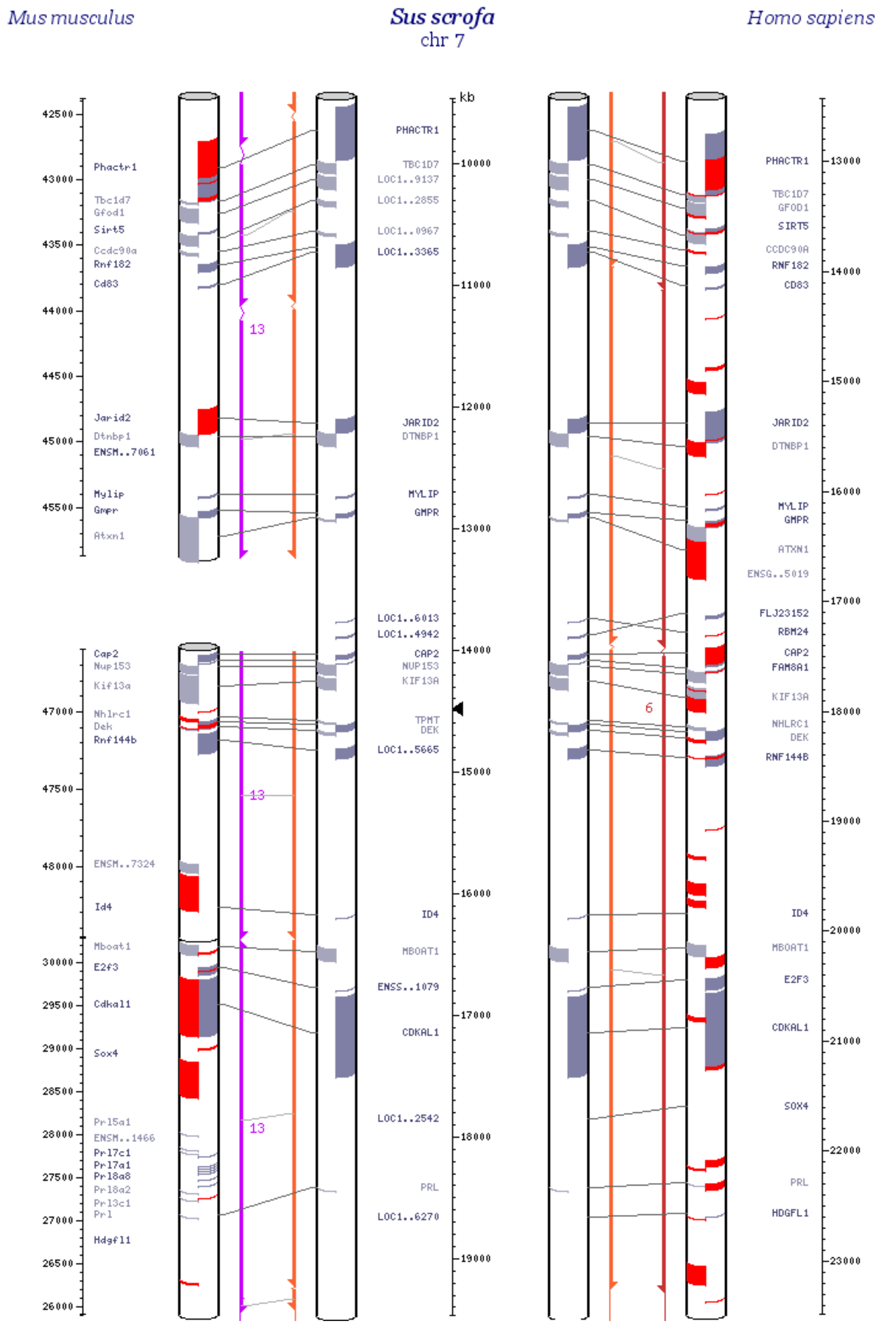

SUPPLEMENTARY FIGURE 13 – The phastCons scores predicted the most conserved elements, and phyloP scores predict a region of conversation of *XLOC_165754* across multiple genomes.

Conservation of *XLOC_165754* in multi species genome, the top of the figure marked as arrow is exon region of *XLOC_165754,* while the region without arrow is the intron of *XLOC_165754,* and the direction of the arrow represents the direction of *XLOC_165754* transcription; ‘PhastCons most conserved’ is the prediction of the most conservative elements based on PhastCons value in multi species sequences; ‘PhastCons post prob‘ is the PhastCons scores in each genome site. ‘PhyloP is score’ means the PhyloP scores in each genome site, it represents the mutation probability of that locus.

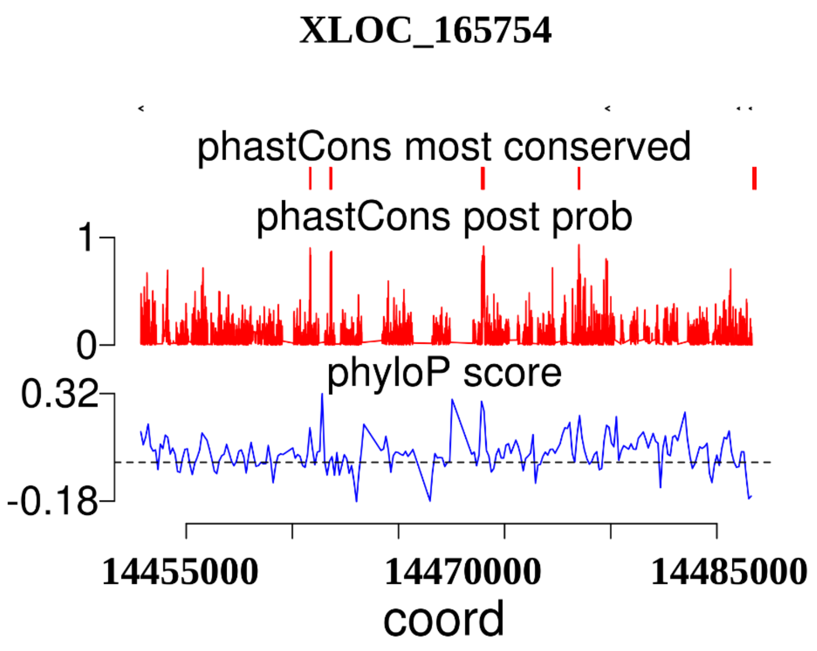

SUPPLEMENTARY FIGURE 14 – ChIP-seq results of histone modification and pluripotent transcription factors for the region of *XLOC_165754*

The bottom of the figure is genomic loci of *XLOC_165754*, the black blocks represent the exons of long non-coding of RNA, and the arrow is the direction of long non-coding RNA transcription; histone epigenetic modification including H2A.Z, H3K27ac, H3K27me3, H3K36me3, H3K4me1/2/3 and H3K9me3, transcription factors including Nanog and Oct4, transcriptional coactivator p300 and negative control of ChIP-seq is IgG.

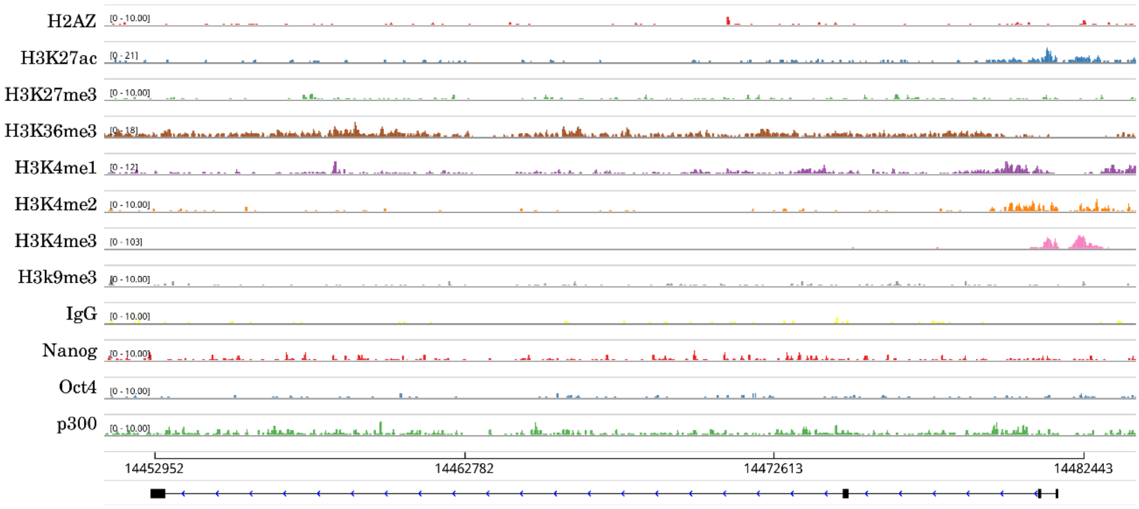

XLOC_165754
